# Lipidome Remodeling and Autophagic Respose in the Arachidonic-Acid-Rich Microalga *Lobosphaera incisa* Under Nitrogen and Phosphorous Deprivation

**DOI:** 10.3389/fpls.2020.614846

**Published:** 2020-11-27

**Authors:** Kamilya Kokabi, Olga Gorelova, Boris Zorin, Shoshana Didi-Cohen, Maxim Itkin, Sergey Malitsky, Alexei Solovchenko, Sammy Boussiba, Inna Khozin-Goldberg

**Affiliations:** ^1^The Albert Katz International School for Desert Studies, The Jacob Blaustein Institutes for Desert Research, Ben-Gurion University of the Negev, Midreshet Ben-Gurion, Israel; ^2^Microalgal Biotechnology Laboratory, The French Associates Institute for Agriculture and Biotechnology of Drylands, The J. Blaustein Institutes for Desert Research, Ben-Gurion University of the Negev, Sede Boqer Campus, Midreshet Ben-Gurion, Israel; ^3^Department of Bioengineering, Faculty of Biology, Moscow State University, GSP-1, Moscow, Russia; ^4^Metabolic Profiling Unit, Life Science Core Facilities, Weizmann Institute of Science, Rehovot, Israel; ^5^Institute of Natural Sciences, Derzhavin Tambov State University, Tambov, Russia; ^6^Peoples Friendship University of Russia (RUDN University), Moscow, Russia

**Keywords:** autophagy, LC-PUFA, lipid, microalgae, nutrient deprivation, triacylglycerol

## Abstract

The green microalga *Lobosphaera incisa* accumulates triacylglycerols (TAGs) with exceptionally high levels of long-chain polyunsaturated fatty acid (LC-PUFA) arachidonic acid (ARA) under nitrogen (N) deprivation. Phosphorous (P) deprivation induces milder changes in fatty acid composition, cell ultrastructure, and growth performance. We hypothesized that the resource-demanding biosynthesis and sequestration of ARA-rich TAG in lipid droplets (LDs) are associated with the enhancement of catabolic processes, including membrane lipid turnover and autophagic activity. Although this work focuses mainly on N deprivation, a comparative analysis of N and P deprivation responses is included. The results of lipidomic profiling showed a differential impact of N and P deprivation on the reorganization of glycerolipids. The formation of TAG under N deprivation was associated with the enhanced breakdown of chloroplast glycerolipids and the formation of lyso-lipids. N-deprived cells displayed a profound reorganization of cell ultrastructure, including internalization of cellular material into autophagic vacuoles, concomitant with the formation of LDs, while P-deprived cells showed better cellular ultrastructural integrity. The expression of the hallmark autophagy protein ATG8 and the major lipid droplet protein (MLDP) genes were coordinately upregulated, but to different extents under either N or P deprivation. The expression of the Δ5-desaturase gene, involved in the final step of ARA biosynthesis, was coordinated with *ATG8* and *MLDP*, exclusively under N deprivation. Concanamycin A, the inhibitor of vacuolar proteolysis and autophagic flux, suppressed growth and enhanced levels of ATG8 and TAG in N-replete cells. The proportions of ARA in TAG decreased with a concomitant increase in oleic acid under both N-replete and N-deprived conditions. The photosynthetic apparatus’s recovery from N deprivation was impaired in the presence of the inhibitor, along with the delayed LD degradation. The GFP-ATG8 processing assay showed the release of free GFP in N-replete and N-deprived cells, supporting the existence of autophagic flux. This study provides the first insight into the homeostatic role of autophagy in *L. incisa* and points to a possible metabolic link between autophagy and ARA-rich TAG biosynthesis.

## Introduction

Microalgae constitute an extremely diverse group of predominantly photosynthetic and aquatic eukaryotic microorganisms with immense biotechnological potential and ecological significance. Dwelling in ever-changing, often harsh, environments, microalgae dynamically respond to variations in nutrient availability. To mitigate nutrient starvation conditions, microalgae arrest their growth, enter a quiescent state, and/or form resilient resting forms for long-term endurance while accumulating storage compounds (Boussiba, 2000; Hu et al., 2008; Dong et al., 2013; Takeuchi and Benning, 2019). Oleaginous species accumulate carbon- and energy-rich reserves in the form of triacylglycerols (TAGs). The production of TAG can be manipulated and enhanced by environmental cues and stress stimuli, such as light and nutrient availability, often by nitrogen (N) deprivation (Hu et al., 2008). Stress-induced TAG accumulation is associated with intensive transcriptional reprograming, metabolome reorganization, membrane lipid remodeling (Hockin et al., 2012; Valledor et al., 2014; Abida et al., 2015; Remmers et al., 2018), and an enhancement in the autophagic response (Martin et al., 1976; Davey et al., 2014; Schatz et al., 2014; Pérez-Pérez et al., 2017; Couso et al., 2018). Under N deprivation, self-cannibalization, scavenging, and recycling of cellular components fuel TAG formation with energy and nutrient requirements. The ultrastructural manifestations of chloroplast degradation, a reduction in cytoplasmic ribosome abundance, and the formation of autophagic vacuoles in the course of lipid droplet (LD) formation were observed in microalgae under various stresses (Zhao et al., 2014; Shebanova et al., 2017; Tsai et al., 2017; Gorelova et al., 2018). Phosphorous (P) deprivation is mitigated by an array of mechanisms, which include membrane lipid remodeling in favor of non-phosphorous lipids, reallocation of cellular C resources, P scavenging from endogenous reserves, and autophagy (Feng et al., 2015; Cañavate et al., 2017; Alipanah et al., 2018; Solovchenko et al., 2019).

Autophagy is a highly conserved and regulated recycling process by which undesired proteins and damaged organelles are degraded in lysosomes (animals) or vacuoles (yeast and plants) to maintain cell homeostasis, survival, and turnover of cellular constituents (see Feng et al., 2014), among others. Recent insights into the mechanisms of autophagy in land plants and photosynthetic microalgae have revealed some distinct features, compared with the process in mammals and yeast (Masclaux-Daubresse et al., 2020). Two major forms of autophagy, macro- and micro-autophagy, have so far been characterized in plants. The analysis of the mutants of the key autophagy-related genes (ATG) has revealed a number of physiological functions attributed to autophagy in the sensing and mobilization of nutrients in plants (Phillips et al., 2008; Avin-Wittenberg et al., 2015; Minina et al., 2018; Chen et al., 2019).

The stress-induced and autophagy-mediated effects in microalgae are of both fundamental and biotechnological importance (Couso et al., 2018; Yoshitake et al., 2019; Kajikawa and Fukuzawa, 2020). The dynamic nature of the micro-algal metabolism, as well as the ways to manipulate it for industrial applications, has attracted significant attention to the study of autophagy’s role. Many tools to study autophagy and monitor autophagic flux have been developed and applied in the model microalga *Chlamydomonas reinhardtii*, illuminating the role of autophagy in cell homeostasis under various stresses, including N and P deprivation (Pérez-Pérez et al., 2016; Couso et al., 2018), among others. The isolation of the first *Chlamydomonas* autophagy mutants, lacking ATG8 and ATG3, marked an important milestone in assessing the function of autophagy in response to nutrient stress by genetic means (Kajikawa et al., 2019). Depleting a hallmark autophagy protein, ATG8, reduced cell viability under N deprivation, and, to a lesser extent, under P and S deprivation. Furthermore, autophagy appeared to be important in the early events of TAG mobilization (Kajikawa et al., 2019). Linking chloroplast quality control, autophagy, and lipid metabolism, the pharmaceutical inhibition of fatty acid synthesis promoted chloroplast damage and triggered autophagy (Heredia-Martínez et al., 2018). Further, administration of the vacuolar ATPase inhibitor concanamycin A, which blocks autophagic flux by repressing autophagy-mediated protein degradation, reduced TAG and LD formation in N-starved *C. reinhardtii* (Couso et al., 2018). Alternatively, the induction of autophagy by pharmaceutically inactivating the target of the rapamycin (TOR) kinase (Imamura et al., 2015; Prioretti et al., 2017), or by inhibiting III PI3K VPS34 that acts in autophagy (Zienkiewicz et al., 2020), induced LD formation and enhanced TAG production in the nutrient-replete Chlorophyte and Stramentopile microalgae.

The mechanisms involved in LD formation, mobilization, and degradation are still understudied in microalgae, in particular, the involvement of autophagy. The dual role of autophagy to reciprocally support LD formation and degradation in plants and microalgae has been proposed (Fan et al., 2019; Tran et al., 2019). The autophagic degradation of LDs under carbon starvation was observed in the unicellular charophyte *Micrasterias denticulata* (Schwarz et al., 2017). Autophagy inhibitors were shown to impair LD remobilization upon nutrient resupply to N-deprived microalgae (Nonoyama et al., 2019; Zienkiewicz et al., 2020). However, further studies in diverse species are obviously needed to clarify whether LDs can be degraded by autophagy-dependent (lipophagy) or independent mechanisms (lipolysis), or both, as suggested in other systems (Schulze et al., 2017).

The present study emerged from our previous work, which reported differential physiological and metabolic responses of the green oleaginous microalga *Lobosphaera incisa* to N and P deprivation. This chlorophyte uniquely accumulates TAG with a high content of the omega-6 long-chain polyunsaturated fatty acid (LC-PUFA) arachidonic acid (ARA, 20:4*n*-6) in the cytoplasmic LDs, under N deprivation. The enhanced biosynthesis and sequestration of ARA in reserve lipids require intensive metabolic expenditures (Kokabi et al., 2019). Upon nutrient replenishment, N-starved cells of *L. incisa* degrade LDs, hydrolyze TAG and remobilize a fraction of ARA-rich TAG to rapidly restore the unsaturation level of chloroplast membrane lipids (Khozin-Goldberg et al., 2005). The assortment of TAG lipases (Siegler et al., 2017), associated with LDs in N-starved *L. incisa*, has recently been identified, presumably contributing differentially to TAG lipolysis under N deprivation or repletion. Although this work mainly focuses on N deprivation, we combined ultrastructural, lipidomics, and some biochemical analyses to comparatively assess some aspects of N and P deprivation responses. We suggested that ARA-rich TAG biosynthesis involves the concerted deployment of catabolic activities to support the metabolic costs associated with ARA biosynthesis and endogenous resource repurposing. Here, we provide several pieces of evidence on the involvement and importance of autophagy in cellular homeostasis and, in general, in ARA-rich TAG accumulation under N deprivation, in particular.

## Materials and Methods

### Algal Strain and Experimental Conditions

*Lobosphaera incisa* (SAG 2468) was cultivated in a modified BG-11 medium in a semi-continuous mode in 250-ml glass flasks placed in an illuminated incubator shaker (New Brunswick G-25, Edison, NJ, United States) in a CO_2_-enriched atmosphere at 25°C, as previously described (Kokabi et al., 2019). For nutrient-deprivation experiments, the cells from regularly diluted replete cultures were collected by centrifugation, washed twice with double-distilled water, and resuspended in mBG-11 lacking either a N source (sodium nitrate) or a P source (K_2_HPO_4_ and KH_2_PO_4_) to an initial chlorophyll content of 30 mg l^−1^ (approximately 1 mg l^−1^ dry weight). Control cultures were grown in complete mBG-11. For recovery from N and P deprivation, cells were collected by centrifugation after 10 days of cultivation, washed, and resuspended in nutrient-replete mBG-11 to an initial chlorophyll content of 15 mg l^−1^. For nutrient deprivation experiments, cells were withdrawn at the logarithmic stage and resuspended in N-depleted or P-depleted mBG-11 media. When indicated, the cultures were supplemented with concanamycin A (Cayman) from a stock solution in DMSO (1 mM); an equal volume of DMSO was added to the control cultures serving as the vehicle control.

### Determination of Fatty Acid Composition and Content

Fatty acid composition and content were determined following direct transmethylation of freeze-dried biomass and isolated TAG in dry methanol containing 2% (v/v) sulfuric acid at 80°C for 1.5 h under an argon atmosphere, using heptadecanoic acid 17:0 as an internal standard. Fatty acid methyl esters (FAME) were resolved by gas chromatography on a Trace GC ultra (Thermo Fisher Scientific, Milan, Italy) equipped with a flame-ionization detector (FID; Pal-Nath et al., 2017).

### Lipid Extraction and TAG Analysis

Lipids were extracted from lyophilized biomass, and total lipid extracts were resolved by one-dimensional thin-layer chromatography TLC on silica-gel plates (Silica Gel 60, 10 × 10 cm, 0.25-mm thickness, Merck, Darmstadt, Germany) to isolate TAG. Extraction, analysis of TAG, and fatty acid quantification were performed by GC-FID (Zorin et al., 2017).

### Lipidomics Profiling by LC-MS

Biomass samples were extracted with a methanol:methyl tert-butyl ether (MTBE) mixture as described by Malitsky et al. (2016), containing the following internal standards: 0.1 μg ml^−1^ phosphatidylcholine 34:0 (17:0/17:0), 0.1 μg ml^−1^ phosphoethanolamine 34:0 (17:0/17:0), 0.15 nmol ml^−1^ of LM6002, and LM6005 d5TG (Merck) standard mix. Post-extraction, the organic phase, containing lipids, was analyzed using a Waters ACQUITY UPLC system coupled to a Vion IMS QTof mass spectrometer (Waters Corp., MA, United States). Chromatographic conditions were as described by Malitsky et al. (2016) with small modifications. Briefly, the chromatographic separation was performed on an ACQUITY UPLC BEH C8 column (2.1 × 100 mm, i.d., 1.7 μm; Waters Corp., MA, United States). Mobile phase A consisted of 45% water (UPLC grade) with 1% 1 M NH_4_Ac, 0.1% acetic acid, and 55% acetonitrile:isopropanol (7:3) with 1% 1 M NH_4_Ac, 0.1% acetic acid (mobile phase B). The column was maintained at 40°C, and the mobile phase flow rate was 0.4 ml min^−1^. Mobile phase A was run for 1 min at 100%; then, it was gradually reduced to 25% at 12 min, followed by a decrease to 0% at 16 min. Then, mobile phase B was run at 100% for 21 min, and mobile phase A was set to 100% at 21.5 min. Finally, a column was equilibrated at 100% of mobile phase A for 25 min. The MS parameters were as follows: the source and de-solvation temperatures were maintained at 120 and 450°C, respectively. The capillary voltage was set at 3.0 and 2 kV for the positive and negative ionization modes, respectively; cone voltage was set at 40 V. Nitrogen was used as a de-solvation gas and cone gas at the flow rates of 800 and 30 L h^−1^, respectively. The mass spectrometer was operated in the full scan MS^E^ positive resolution mode over a mass range of 50–1800 Da. For the high energy scan function, a collision energy ramp of 20–60 eV was applied; for the low energy scan function, −4 eV was applied. Leucine-enkephalin was used as a lock-mass reference standard.

### Lipid Species Identification and Relative Normalization

LC-MS data were analyzed and processed with UNIFI (Version 1.9.2, Waters Corp., MA, United States). The putative identification of the different lipid species was performed by a comparison of accurate mass, fragmentation pattern, and ion mobility (CCS) values with an in-house-made lipid database, in which several lipids were identified vs. standards, when available. Further annotation of the lipids was based on the correlation between the retention time (RT) and the carbon chain length and degree of unsaturation. Validation of the putative lipid identification was performed by a comparison with a home-made library, which contains lipids produced by various organisms. Relative lipid levels were normalized to the internal standards (see Lipid Species Identification and Relative Normalization) and the amount of tissue used for analysis.

### Electron Microscopy

Cells for TEM were harvested from approximately 5-ml culture aliquots. The fixation, dehydration, embedding, and staining procedures were described by Kokabi et al. (2019). Ultra-thin sections (60–90 nm) were observed and imaged under an FEI Tecnai 12 (FEI, OR, United States) or a JEM-1011 (JEOL, Tokyo, Japan) transmission electron microscope (TEM) for the ultrastructural analysis.

The samples for nanoscale elemental analysis in analytical TEM using EDX were fixed, dehydrated, and embedded in araldite (Electron Microscopy Sciences, United States) as described above. Ultra-thin sections for energy-dispersive X-ray spectroscopy (EDX) analysis were examined under a JEM-2100 (JEOL, Tokyo, Japan) microscope equipped with a LaB_6_ gun at the accelerating voltage of 200 kV. Point EDX spectra were recorded using a JEOL bright-field scanning TEM (STEM) module and an X-Max X-ray detector system with an ultra-thin window, capable of analyzing light elements, starting from boron (Oxford Instruments, High Wycombe, UK). The energy range of the recorded spectra was 0–10 keV with a resolution of 10 eV per channel. Spectra were background-corrected and further processed with INKA software (Oxford Instruments). Quantitative morphometric analysis of TEM micrographs was performed using Zen software (Carl Zeiss Microscopy GmbH).

### Fluorescence and Immunofluorescence Microscopy

Nile Red (NR) staining (Sigma-Aldrich) and imaging of LDs was performed as described by Kokabi et al. (2019). For the immunofluorescence detection of *Li*ATG8, the cells were collected by centrifugation (3,000 rpm for 1 min) from approximately 2-ml aliquots of regularly diluted culture grown in the replete medium. The pellet was fixed with 4% paraformaldehyde in phosphate-buffered saline (PBS) for 20 min at room temperature (RT), and then rinsed three times with PBS. The cells were permeabilized with 1% Triton X-100 in PBS for 10 min at RT. After blocking with 2% bovine serum albumin in PBS for 30 min, cells were labeled at RT for 1 h with anti-ATG8 antibody diluted to 1:200 in a blocking solution. The cells were rinsed three times with PBS and incubated with secondary antibody Alexa Fluor 488 donkey anti-rabbit IgG (Jackson IR Laboratories) at a dilution of 1:500 for 40 min. After washing three times, cells were observed under a Zeiss LSM 880 Axio Observer laser scanning confocal microscope using an alpha Plan-Apochromat 63×/1.4 Oil DIC M27 objective. Images were analyzed by Zen Blue software (Zeiss).

### RNA and DNA Isolation, cDNA Synthesis, and Quantitative Real-Time PCR

Total RNA was isolated from the cell pellets placed in 2-ml Eppendorf safe-lock tubes and snap-frozen in liquid N_2_. Frozen cells were disrupted by using 2.5-mm iron beads in a Retsch MM400 tissue homogenizer in 7–8 cycles of 45 s at 25 Hz, and placing tubes in liquid N_2_ between cycles. An SV Total RNA isolation kit (Promega, Madison, WI, United States) was used, according to the manufacturer’s protocol. The genomic DNA isolation was performed as described by Zorin et al. (2014). cDNA was synthesized by a Verso cDNA synthesis kit (Thermo Fisher Scientific) on a 500-ng aliquot of total RNA pre-heated at 70°C for 5 min to eliminate secondary structures. The qRT-PCR was performed with iTaq Universal SYBR Green Supermix (Bio-Rad Laboratories, Hercules, CA, United States) on a CFX96 Touch Real-Time PCR Detection System (Bio-Rad, Hercules, CA, United States). Genes that were constitutively expressed under N starvation were used as reference genes after confirming their stable expression. The primers used in this study are listed in Supplementary Table S1. The primers used in qRT-PCR were tested for amplification efficiency (95–100%) by generating calibration curves. Gene expression was quantified by the ∆∆Cq method in CFX Manager Software (Bio-Rad).

### Plasmid Construction for GFP-ATG8 Tagging

To create the N-terminal fusion of GFP to the ATG8 gene, the entire genomic sequence of ATG8, including the promoter (269 bp) and terminator (508 bp) regions, was amplified by PCR using a Phusion High-Fidelity DNA-polymerase (BioLAbs New England, United States). The primers were designed to introduce *SpeI* restriction sites for further manipulation (Supplementary Table S1). The PCR product of expected size was purified from agarose using a Zymoclean Gel DNA Recovery Kit and inserted into the pJet-AHAS plasmid using isoschizomer *XbaI* and *SpeI* enzymes. The plasmid contained the mutated sequence of the *L. incisa* acetohydroxyacid synthase (AHAS) gene, conferring resistance to the herbicide sulfometuron methyl (SMM), as an endogenous selection marker (Grundman et al., 2012). The PCR-amplified codon-optimized eGFP (Genescript) without a stop codon and the linearized vector with the inserted *Li*ATG8 gene were obtained with 15-bp overlaps at their 5' and 3' ends. The fragments were ligated using an In-Fusion HD Cloning Kit (Takara Clontech, United State) according to the manufacturer’s protocols. *Escherichia coli* strain Zymo 5α competent cells were used for cloning, and transformation procedures were performed with a Mix & Go Kit (Zymo Research, United States). Transformed colonies were selected based on ampicillin resistance and examined for the presence of inserts using an on-colony PCR. Plasmids were purified from *E. coli* cultures using a Midi Plasmid Kit (Geneaid Biotech Ltd., Taiwan).

### Nuclear Transformation of *L. incisa*

The nuclear transformation was performed by electroporation using a Gene Pulser XCell apparatus (Bio-Rad, CA, United States) as described by Zorin et al. (2014). Transformed cells were plated on modified LB agar plates, supplemented with 30 μM SMM and grown at 25°C and a light intensity of 35 μmol photons m^−2^ s^−1^. After the second sub-culturing under selection pressure, transgene integration was verified by PCR.

### Detection of the GFP-Tagged *Li*ATG8 Protein by Confocal Microscopy

Confocal microscopy was conducted using a Zeiss LSM 510 Meta confocal microscope equipped with AIM 4.2 software. In order to visualize the GFP signal in *L. incisa* cells, we used a Plan-Apochromat 63×/1.4 oil objective with an argon laser (488 nm). To diminish the background and chlorophyll autofluorescence, the signal was collected using a 505/50 emission filter. Image analysis was conducted using Zeiss LSM Image Examiner (Version 3.5.0.359) software.

### Protein Preparation and Western Blot Analysis

The culture aliquots of WT *L. incisa* or transformants expressing GFP-ATG8 were collected by centrifugation at 3,000 rpm for 5 min. The pellet was washed twice with double-distilled water (DDW) and stored at −80°C for further use. The total protein extraction was performed from 2 to 5 mg of dry weight (DW) by grinding frozen biomass in liquid nitrogen with a mortar and pestle with a minimal amount of extraction buffer [1.25 mM EDTA, 250 mM Tris-HCl pH 7.5, 250 mM sucrose, 4 mM dithiothreitol (DTT), 14.3 mM glutathione (GSH), and 1 × protease inhibitor cocktail (P9599; all reagents were from Sigma-Aldrich]. The homogenate was centrifuged at 13,000 rpm for 15 min at 4°C, and the protein content in the supernatant was estimated by Bradford assay (Bio-Rad). Western blot analysis was performed on the protein extracts of the samples collected at the specified time points, using the primary anti-CrATG8 (Agrisera) or anti-GFP (Evrogen, Russia) antibodies. Either 2 or 5 μg of total proteins was loaded into each well and separated on a 15 or 10% SDS-PAGE gel for detection of ATG8 or GFP, respectively. The proteins were transferred from the gel onto a nitrocellulose 0.2-μm membrane (BioRad) for 45 min at 120 V. The membrane was incubated with 5% nonfat milk in 1 × phosphate-buffered saline (PBS) with 0.5% Tween-20 (PBS-T) for 1 h at room temperature (RT), and then incubated with either an anti-CrATG8 or anti-GFP antibody with 1:1,000 dilution at 4°C overnight. The membrane was washed three times with PBS-T and incubated with an anti-rabbit secondary (BioRad) antibody at 1:10,000 dilutions for 1 h at room temperature. The blot was washed three times and developed with the chemiluminescence detection kit for HRP (EZ-ECL Kit, Biological Industries, BI-20-500-120) according to the manufacturer’s protocols.

### Statistical Analysis

Student’s *t*-test was used to determine statistically significant differences. Significance was determined at *p* < 0.05. The webserver MetaboAnalyst 4.0 (Chong et al., 2018) was used for statistical analysis of lipidomics data to compare changes in lipid species among treatments. The data were filtered by relative standard deviation (RSD = SD/mean), auto-scaled, log-transformed, and normalized. Significant differences were determined using ANOVA with a Tukey *post hoc* test. FDR adjusted *p* < 0.05 were used to account for multiple comparisons.

## Results

### Ultrastructural Modifications in the Course of N and P Deprivation

**Figure 1:**
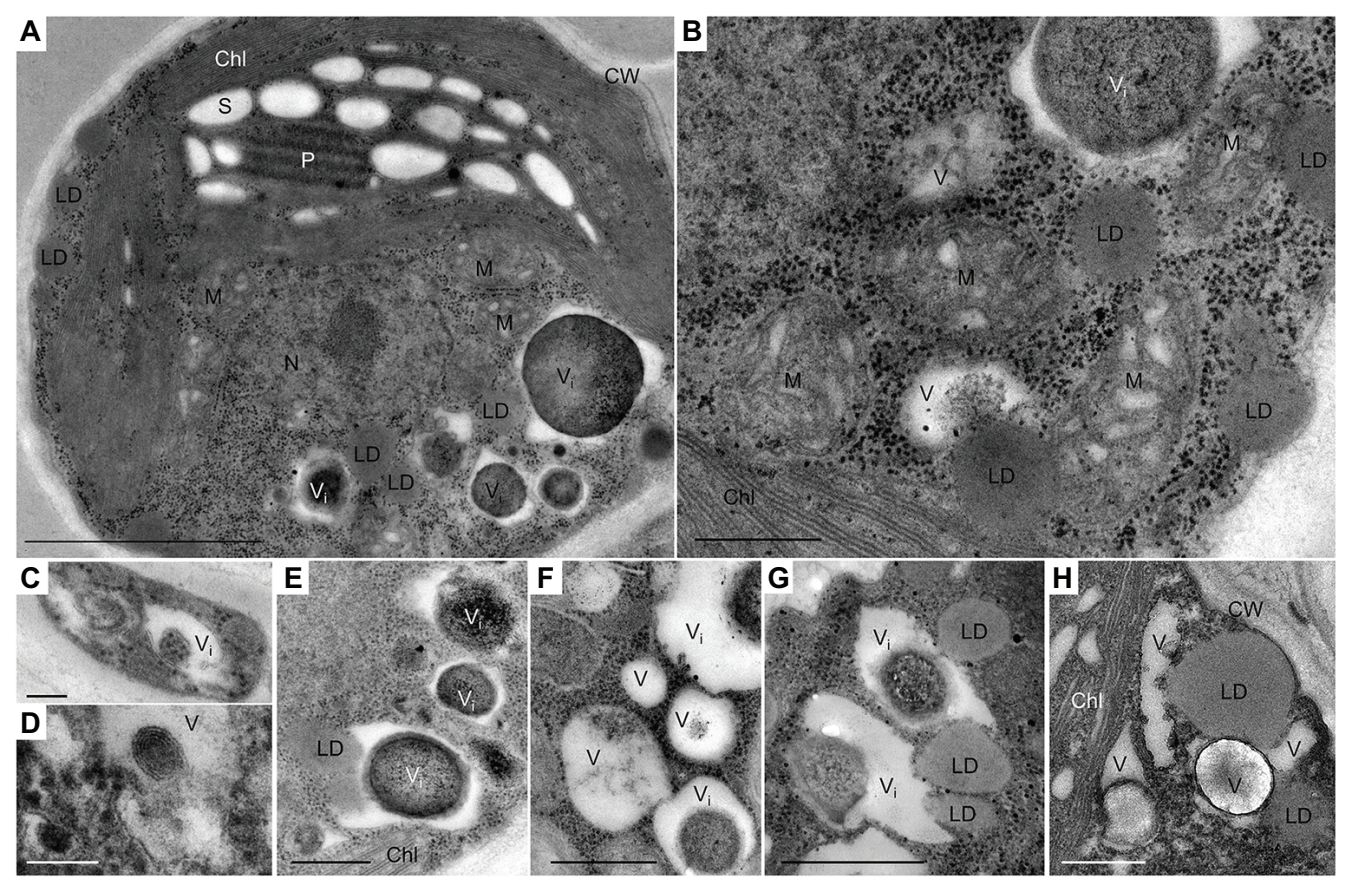
Transmission electron micrographs of *L. incisa* cells in the logarithmic growth stage. **(A)** General cell ultrastructure and **(B)** different cytoplasmic components; **(C–H)** vacuoles and vacuole-like structures with different inclusions. LD-vacuole interactions, as indicated by their close special association (**E,G,H)**. Chl, chloroplast; S, starch granules; CW, cell wall; LD, lipid droplet; P, pyrenoid; M, mitochondria; N, nucleus; V, vacuole; Vi, vacuole with electron-dense inclusion. Scale bars in **(A)** = 1 μm, **(B,E–H)** = 0.5 μm, and **(C,D)** = 0.1 μm.

In our prior work, some ultrastructural features of *L. incisa*, under nutrient-replete conditions and after 3 days of N and P deprivation have been described (Kokabi et al., 2019). Here, using TEM, we mainly focused on the dynamics of vacuoles and vacuole-like structures, including their inclusions and interactions with LDs under N deprivation and following nutrient resupply. The replete cells of *L. incisa* harbor a variety of acidic vacuoles and vacuole-like structures of diverse sizes and shapes, containing various inclusions, predominantly polyphosphate (polyP), and N-containing granules (Kokabi et al., 2019; Figures 1A,B). We assumed that the abundant organelles housing polyP granules may mirror the acidocalcisomes described in microorganisms and microalgae (Goodenough et al., 2019). We also observed vacuoles lacking polyP granules, for instance, sequestering the portions of the cytoplasm with ribosomes in young daughter cells (Figure 1C), harboring the multiple twisted-strand structures (Figure 1D), or including non-homogeneous electron density (Figures 1E–H). Notably, the small cytoplasmic LDs in N-replete cells were often located in close proximity to the vacuoles, and even seemed to be partially engulfed by them (Figures 1A,B,E,G,H).

**Figure 2:**
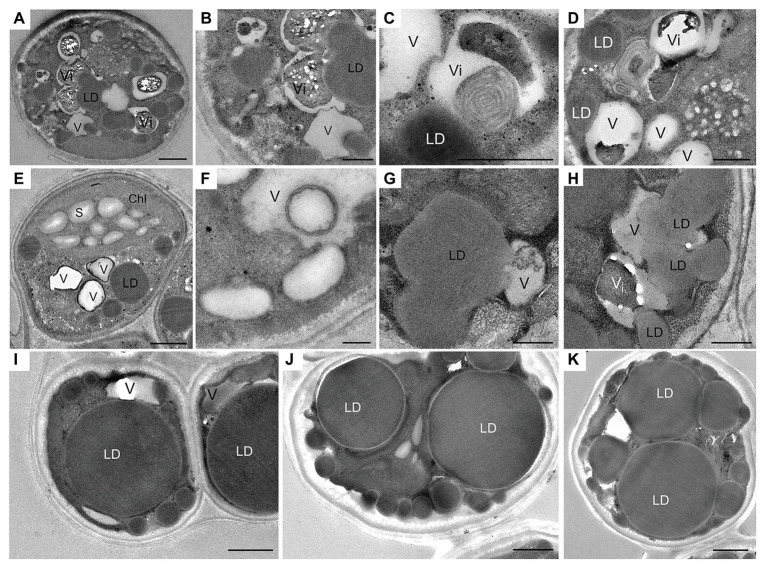
Ultrastructural changes in *L. incisa* cells under N deprivation: **(A–D)** N-deprived cell after 12 h; **(E,F)** 24 h; **(G,H)** 48 h; **(I−K)** 10 days. LD, lipid droplet; V, vacuole; Vi, vacuole with electron-dense inclusion; Chl, chloroplast; S, starch granules. Scale bars in **(A,I,J,H)** = 1 μm and **(B–G,K)** = 0.5 μm.

The cell ultrastructure was drastically altered after exposure to N deprivation. The main changes, 12 h following the transfer to the N-deprived medium, included the fusion of vacuoles (Figure 2A), the partial degradation of granular bodies in vacuole interiors (Figures 2A–D), and the increased diversity of vacuolar inclusions (Figures 2B–D). Vacuoles with membranous debris visible as multi-lamellar structures were observed. Similarly, autophagic vacuoles, with various cargo material and debris have been described in N-starved *C. reinhardtii* (Goodenough et al., 2014), *Dunaliella salina* (Polle et al., 2020), and Cd^2+^-stressed *Micrasterias denticulata* (Andosch et al., 2012). Autophagy-related vacuoles, containing membrane debris, can be distinguished from acidocalcisomes using advanced microscopic methods, such as quick-freeze deep-etch electron microscopy, and their appearance generally coincides with the expression of genes associated with autophagy (Goodenough et al., 2014, 2019).

**Figure 3:**
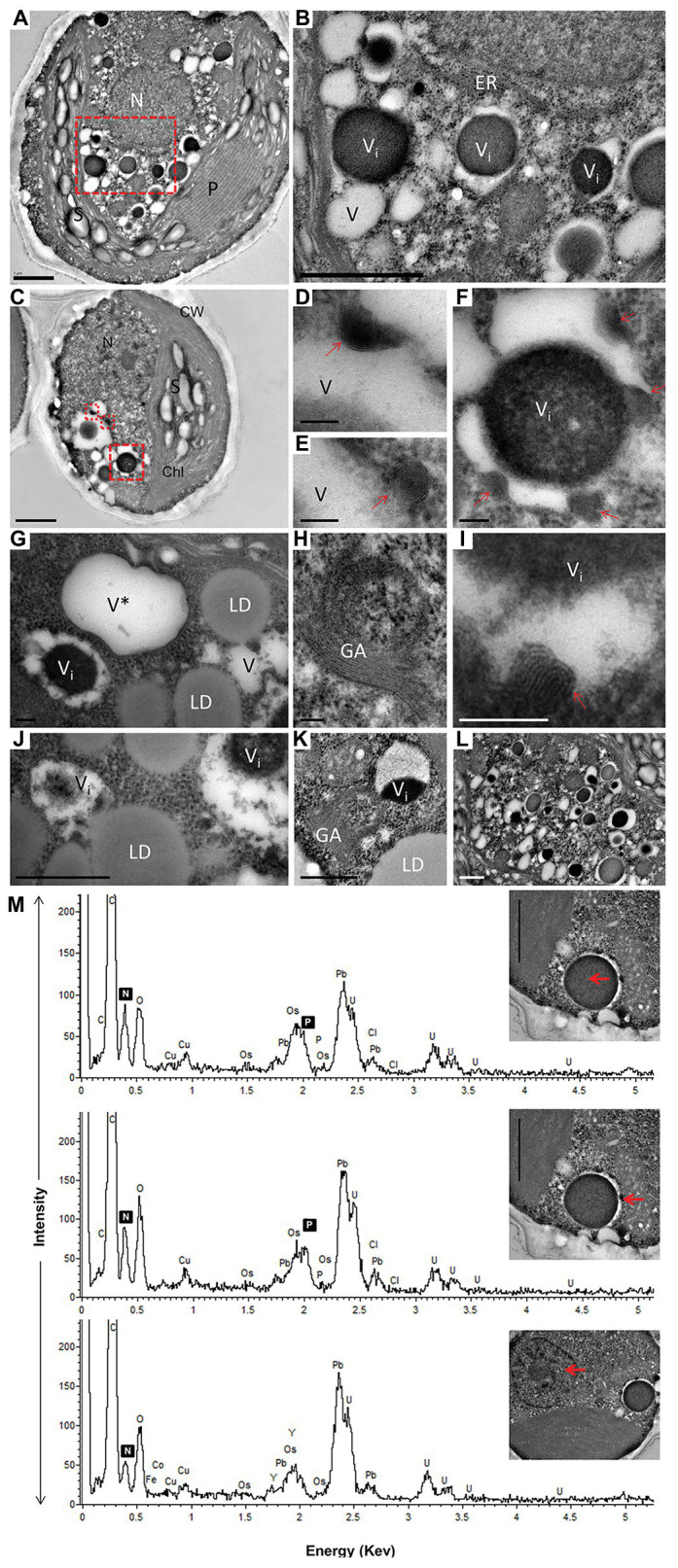
TEM image **(A–L)** with elemental EDX analysis **(M)** of P-deprived cells for 3 days. The squares with red broken lines in **(A,C)** demark an area shown at higher magnification in **(B,D–F)**, respectively. Elemental EDX point spectra on vacuolar inclusions and nucleus **(M)** recorded in a STEM-mode on ultra-thin sections. Chl, chloroplast; S, starch granules; LD, lipid droplets; P, pyrenoid; N, nucleus; GA, Golgi apparatus; ER, rough endoplasmic reticulum; V, vacuole; V*, vacuole with a double membrane; **(D–F,I)** multiple twisted strands connected to the tonoplast (red arrow) in the vacuole; **(G,J)** close contact of LD with vacuole. Scale bars in **(A–C)** = 1 μm, **(J–L)** = 0.5 μm and **(D–I)** = 0.1 μm.

Numerous cytoplasmic LDs were formed with the progression of N deprivation, while the vacuole abundance decreased (Table 1; all vacuole-like structures were considered). During their expansion and growth, LDs frequently showed a close special association with vacuoles and even seemed to appear inside the vacuolar lumen (Figure 2). After 10 days of N deprivation, LDs occupied over 50% of the cell volume; starch granules were observed near the partially lysed pyrenoid in the abridged chloroplast (not shown), while the vacuoles became fewer and empty, and the abundance of cytoplasmic components decreased (Figure 2I). N deprivation led to more severe growth retardation than P deprivation and was associated with a concomitant decline in the photosynthetic pigment content and chloroplast dismantling (Kokabi et al., 2019). Indeed, P-deprived cells (Figure 3) displayed fewer LDs and a far better integrity of the chloroplast and cytoplasmic components. A distinct feature of P-deprived cells was the structure of vacuolar inclusions, showing multiple twisted strands connected to the tonoplast (Figure 3), presumably consisting of polyP chains combined with N-containing material (Figure 3M).

**Table 1:** Morphometric analysis of vacuoles and lipid droplets in nutrient-replete and N-deprived (2 days-N) *L. incisa* cells.

Time point	Vacuole per cell		LD per cell	
	Number	Diameter (μm)	Number	Diameter (μm)
Time 0	4.6 (±0.28)	0.80 (±0.04)	2.5 (±0.36)	0.31 (±0.05)
2d-N	3.05 (±0.23)	0.82 (±0.08)	10.7 (±0.31)	0.78 (±0.06)

Quantification analysis was carried out from 20 TEM images at each time point.

**Figure 4:**
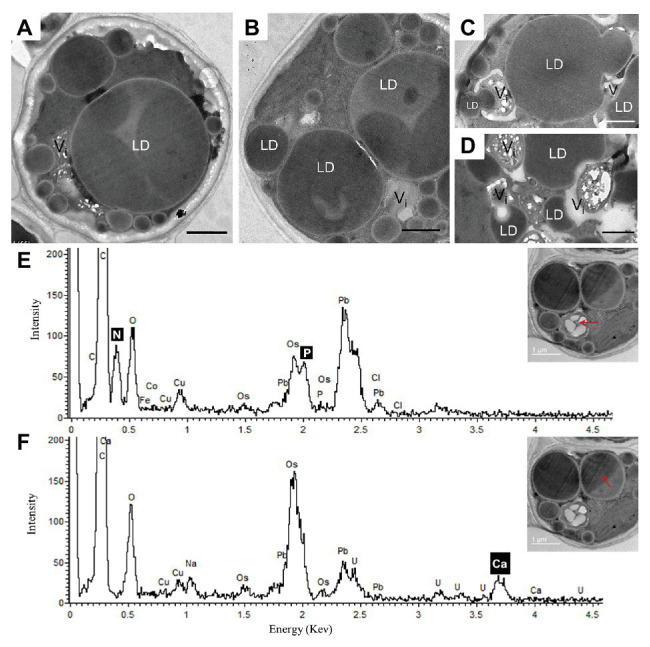
Ultrastructural changes in *L. incisa* after nutrient replenishment to N-deprived cells (24 h) **(A–D)**. Elemental EDX point spectra on degrading lipid bodies and vacuolar inclusions **(E,F**) recorded in a STEM-mode on ultra-thin sections. LD, lipid droplet; V, vacuole. Scale bars in **(A–F)** = 1 μm.

After 10 days of N deprivation, nutrients were replenished by resuspending the cells in the replete medium, and cells were observed at TEM. After 24 h, a fraction of LDs fragmented into smaller ones (Figure 4). The partial enclosure of LDs with vacuole-like structures and degradation at LD-vacuole contact sites resembled a macroautophagy-like process observed in the oleaginous microalga *Nannochloropsis oceanica* and *Arabidopsis thaliana* leaves (Tsai et al., 2014; Fan et al., 2019). Notably, the core of degrading LDs displayed an inhomogeneous electron density, suggesting compositional transitions in the TAG. Along with an increase in their number, the emerging and initially visibly empty vacuoles filled up with electron-dense material. N and P peaks in the point spectra of vacuolar inclusions were detected by EDX in a STEM mode (Figure 4E), suggesting an acquisition of N and P by vacuoles after nutrient replenishment. Given certain limitations of the EDX analysis on the resin-embedded samples, it was used to distinguish inclusions containing both N and P from those containing only P (in addition to O and C; Ismagulova et al., 2018). EDX did not detect Ca in the vacuoles of replenished cells (as in replete cells), but interestingly, Ca and Na signals were also found to be associated with degrading LDs, regardless of the LD-core osmophilicity (Figure 4F). We speculate that the Ca signal may be due to the LD-associated Ca-binding protein caleosin identified in the *L. incisa* LD proteome (Siegler et al., 2017), and whose possible function in LD degradation will be discussed later. The chloroplast and chlorophyll production recovered by day 2–3, following nutrient resupply (Kokabi et al., 2019; section Pharmaceutical Treatment with Concanamycin A Impairs ARA Biosynthesis).

### Comparative Lipidomics Profiling Under N and P Deprivation

**Figure 5:**
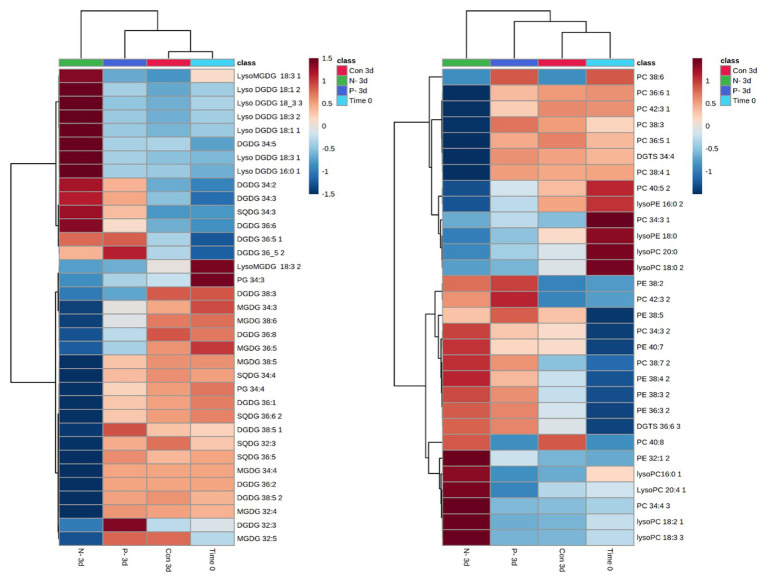
Patterns in chloroplast and extraplastidial glycerolipid changes after 3 days of N and P deprivation compared with time 0 (at the onset of the experiment) and 3 days of nutrient-repletion (Con 3d). The results are shown for the top significantly different species among four conditions ranked by ANOVA, according to the adjusted value of *p* (FDR) cutoff 0.05. Statistical analysis, hierarchical clustering, and heatmaps were performed at https://www.metaboanalyst.ca (Chong et al., 2018). The original data (relative intensities of detected lipid species from four conditions) were quantile-normalized and log-transformed; distance was measured using Pearson’s correlation. Lipid species are designated as C:N, where C is the total number of carbons in acyl chains, and N is the total number of double bonds. MGDG, monogalactosyldiacylglycerol; DGDG, digalactosyldiacylglycerol; DGTS, diacylglyceryl-N,N,N-trimethylhomoserine; PC, phosphatidylcholine; PE, phosphatidylethanolamine; PG, phosphatidylglycerol; SQDG, sulfoquinovosyldiacylglycerol. Positional analysis of acyl group distribution was not performed.

**Figure 6:**
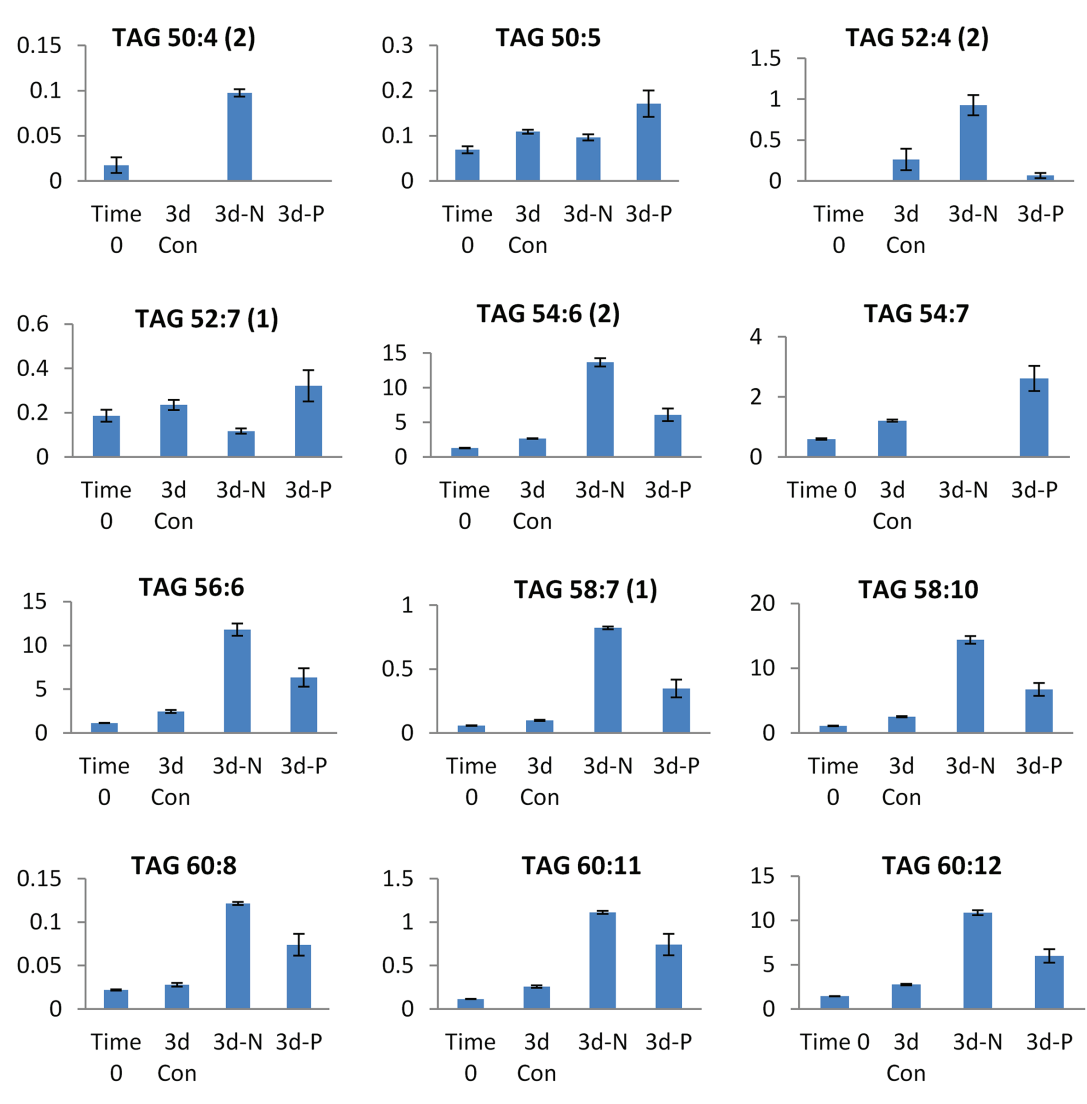
Selected TAG species showing significant differences in their relative abundance (presented in Y-axis) between N and P deprivation conditions. Student’s *t*-test (<0.05) test was applied.

*Lobosphaera incisa*’s fatty acid and glycerolipid composition responded differently to N and P deprivation (Kokabi et al., 2019). In this report, the LC-MS-based lipidomics profiling in a semi-quantitative mode was performed to depict additional features pertaining to the differential impacts of P and N deprivation on the TAG biosynthesis and membrane lipid remodeling. The relative abundance of lipid molecular species was determined using internal standards and normalization to dry weight after 3 days of P and N deprivation, as compared with time 0 and 3 days of nutrient-replete conditions (+N+P). The results showed extensive rearrangements in the profile of structural and storage glycerolipids. The lipidome of N-deprived cells (3d-N) differed significantly from P-deprived (3d−P) and control (3d+N+P) cells. A principal component analysis (PCA), performed on all lipids detected in a negative mode, demonstrated that a high level of variation in the lipidome (58.5%) among conditions can be explained by the first component (PC1; Supplementary Figure S1). To enable comparison among lipid species and conditions, the relative abundance of significantly different species was normalized and visualized using heatmaps (Figures 5, 6).

Similarities and specific nutrient-deprivation differences in the relative abundance of polar and neutral glycerolipids were examined. A distinct pattern of the polar lipidome remodeling under P deprivation, compared with N deprivation, was reflected in the far lesser extent of reorganization (Figure 5). The abundance of most of the detected chloroplast MGDG and SQDG species significantly decreased, while an increase in the number of DGDG species, and in particular, the accumulation of lyso-MGDGs and lyso-DGDGs, constituted a distinctive response to N deprivation. As expected, the chloroplast lipids were less altered under P deprivation. An increase in several PC and DGDG species under P deprivation may suggest the re-assembly of DGDG from PC. Indeed, PC may transiently increase under P deprivation for their reuse in DGDG biosynthesis (Jouhet et al., 2003). Concomitantly with the intensive degradation of chloroplast lipids under N deprivation, a number of lyso-PC species with 16:0, 18:2, 18:3, and 20:4 acyl groups exhibited a marked increase in their abundance. In addition, C20/C18 (38:3, 38:4; 38:5, and 38:7) and C20/C20 (40:7 and 40:8) PE species increased, in agreement with the proposed role of PE in the terminal step of ARA biosynthesis, followed by its shuffling to TAG (Bigogno et al., 2002). PE 36:3, DGTS 36:6, and PC 32:4, 38:7, 40:8 were also more represented in N-deprived cells.

The patterns of changes in the significantly altered TAG species under four experimental conditions are shown in Supplementary Figure S2. The lipidome of N-deprived cells displayed a set of strongly increased TAG species, with only a few of them shared with P-deprived cells. Approximately half of the detected TAG species either increased in a coordinated manner or decreased under N deprivation (Supplementary Figure S2). Among detected TAGs, several mono-, di-, and tri-ARA-containing species (C56, C58, and C60 TAGs) showed a significant increase in their relative abundance under N deprivation compared with P deprivation (Figure 6). Several C18-containing TAG species C56 (18/18/20) and C54 (18/18/18) were more presented in P-deprived cells (as 52:7 and 54:7). Noticeably, the very long-chain and highly unsaturated TAGs (C62 and 64) were detected in this study in *L. incisa*, and several C64 TAG species showed an increasing pattern in P-deprived cells as compared to four conditions examined (Supplementary Figure S2).

Taken together, the results of lipid profiling showed a coordinated but distinct reorganization of polar and neutral lipidomes under P and N deprivation. A stronger decline in membrane glycerolipids and an increase in the ARA-TAG species under N deprivation, compared to P deprivation, were in agreement with the greater extent of chloroplast dismantling, LD formation, and autophagic-like response.

### Identification of Autophagy-Related (ATG) Genes in *L. incisa* and Their Expression in the Transciptome of N-Deprived Cells

Taking into account indications of the autophagic response observed in this and our previous work (Kokabi et al., 2019), we next searched the genome of *L. incisa* (deposited in NCBI under BioProject PRJNA262782 and PRJNA283614; Tourasse et al., in preparation) for the orthologs of autophagy-related proteins (ATG). Details on genomic and transcriptomic sequencing are reported in Siegler et al. (2017).

The putative ATG genes are present in the *L. incisa* genome in a single copy (Supplementary Table S2), and have their closest homologs in Trebouxiophyceae – the major class of green algae (Chlorophyta). Some deduced *L. incisa* ATG-proteins differed in their amino acid length and domain architecture from homologous proteins in other taxa (yeast, mammals, and higher plants) and showed similarity predominantly in the N terminal portion (Supplementary Table S2). WD domains with functions in various cellular processes, such as signaling and vesicle transport, were present in several ATG proteins (Supplementary Table S2). Interestingly, the majority of *ATG* genes are located relatively close to each other in the genome context, for example, *ATG13* (g9857), *ATG5* (g10190), *ATG1* (g10441), *ATG2* (g11309), and *ATG18* (g11431). The genes for *ATG4* (g4008), *ATG3* (g4126), *ATG8* (g4496), and *ATG7* (g5114), involved in the ubiquitin-like conjugation system of the autophagy machinery, are also located nearby in the genome. The function of identified *L. incisa* ATG-proteins in autophagy is hypothetical and requires further study.

The hallmark autophagy protein ATG8 showed high conservation and similarity to ATG8 proteins of other organisms. For instance, it is 83% identical to ATG8 from the model green alga *C. reinhardii*, while sharing 78 and 85% identity with *A. thaliana* ATG8A and ATG8D isoforms, respectively. It shares 74% similarity with the ATG8 of *S. cerevisiae* and is the most similar (98% identity) to a putative ATG8 of *Coccomyxa subellipsoidea* C-169, which belongs to the same class Trebouxiophyceae. The C-terminal cleavage site that is required for ATG8 lipidation is located at G119 (Supplementary Figure S3).

**Figure 7:**
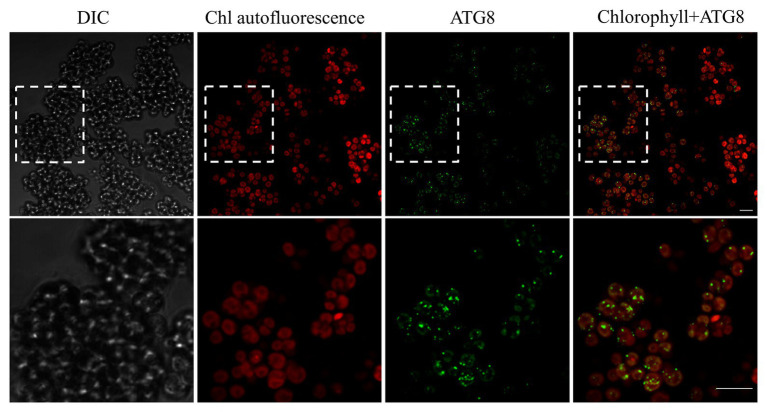
Confocal immunofluorescence micrographs of *L. incisa* cells grown in a replete nutrient medium, displaying the *Li*ATG8 localization as bright green spots in the cytoplasm. Scale bar 10 μm. Cells were processed for immunofluorescence microscopy with an anti-ATG8 primary antibody and Alexa Fluor 488 donkey anti-rabbit IgG secondary antibody. DIC, Differential interference contrast; Chl, (Chlorophyll) autofluorescence; Chlorophyll+ATG8, merge image.

A transcriptomic study of *L. incisa* subjected to N deprivation for 12 and 72 h has been previously performed (Siegler et al., 2017). Among annotated *ATG*-related genes, the expression levels of *ATG1*, *ATG8, ATG14, ATG101*, and *VMP1* (vacuolar membrane protein 1) significantly increased after 72 h of N starvation (Table 2). Remarkably, *ATG8* displayed the highest FPKM values in nutrient-replete cells (day 0), compared with other *ATG*-related genes, and was strongly upregulated in response to N deprivation. In line with the *ATG8* gene expression in replete cells, the immunolocalization of ATG8 in nutrient-replete *L. incisa* revealed several spots per cell in the cytoplasm (Figure 7).

**Table 2:** Significantly upregulated ATG-related genes after 3 days of N deprivation in the transcriptomics study.

Gene ID	Gene symbol	FPKM		Fold change to time 0	*p* adjusted
		0 d	3d-N		
g10441	ATG1	6.96	17.66	2.46	0.001
g4496	ATG8	264.77	827.23	3.53	8e-0.5
g5843	ATG14	10.95	24.30	2.32	0.005
g4696	ATG101	11.11	32.25	3.25	0.0001
g8948	VMP1	13.81	25.93	2.04	0.04

The significance of fold-change is indicated by the *p* value adjusted for multiple testing at a false discovery rate of 0.05. FPKM: Fragments Per Kilobase Million. The L. incisa gene expression data have been deposited in NCBI GEO under accession GSE94666. Conditions and methods of the transcriptomics analysis are described in (Siegler et al., 2017). 0 day, nutrient replete culture; 3d-N, 3 days of N deprivation.

### Expression of Selected Target Genes During N and P Deprivation Followed by Nutrient Replenishment

**Figure 8:**
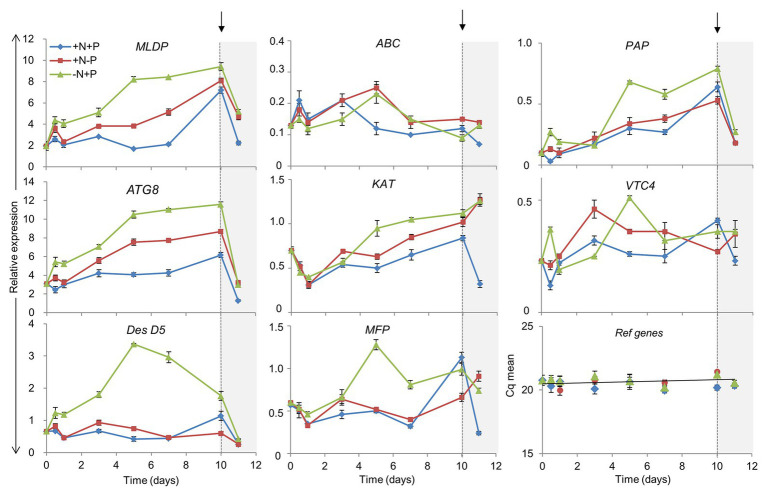
Impact of N and P deprivation on the expression of selected genes related to lipid droplet formation, ARA biosynthesis, fatty acid β-oxidation, polyP metabolism, and autophagy. Control, replete culture (+N+P), blue lines; P deprivation (+N−P), red lines; N deprivation (−N+P), green lines. After 10 days of the indicated conditions, cells were harvested and nutrients replenished by resuspension of the cells in a replete medium: arrows indicate the time of nutrient resupply. Data are presented as means ± SD of the pooled RNA of three biological repetitions (*n* = 3), reverse-transcribed, and analyzed in three technical replicates. For gene abbreviations, refer to the text.

To determine whether differences in the gene expression of several relevant pathways (Supplementary Table S3) could be involved in *L. incisa* responses to N and P deprivation, we monitored the expression levels of selected genes, related to ARA biosynthesis, LD biogenesis, polyP metabolism, fatty acid β-oxidation, and autophagy (Figure 8). The results of a quantitative RT-PCR were normalized to the geometric mean of two reference genes (cytochrome-c oxidase COX3, and small nuclear ribonucleoprotein SNRP), which were stably expressed under experimental conditions.

The Δ5 desaturase mediates the terminal step in the biosynthesis of ARA (Iskandarov et al., 2010). *DesD5* expression displayed a large increase in the initial stages of N deprivation (Figure 8), consistent with previous studies (Iskandarov et al., 2010) and the transcriptomic data (Supplementary Table S3), but remained almost unaltered under P deprivation and in the +N+P control. *DesD5* expression exhibited a drop 24 h after nutrient replenishment under all conditions. The transcripts of the gene encoding the major LD protein (*MLDP*) were substantially and progressively upregulated under N and P deprivation, and in the late stationary phase, in the control culture. *MLDP* and *ATG8* were expressed in concert, in the course of N deprivation: with an over 5-fold increase in the later stages, and a strong decline after nutrient resupply. Under P deprivation, and in the replete cells, *ATG8* and *MLDP* were upregulated, but to a lesser extent, while *DesD5* expression was not altered, in line with the lower ARA production under P deprivation (Kokabi et al., 2019). Given the coordinated expression patterns of the ATG8 and MLDP genes, we searched for ATG8-interacting motifs in the MLDP sequence, using three bioinformatics resources trained on plant and non-plant organisms: iLIR^1^; hfAIM^2^; and ELM.^3^ In *Nannochloropsis oceanica*, the major LD protein LDSP was predicted to possess an ATG8-interacting iLIR motif in silico and was shown to interact with ATG8 *in vivo* (Zienkiewicz et al., 2020). In the MLDP of *L. incisa*, the propensity for MLDP-ATG8 interactions was predicted only by ELM.

Since vacuolar polyP is an important depot of intracellular PO_4_, we monitored the expression of two genes potentially associated with the metabolism of vacuolar polyP. The gene encoding the putative acid phosphatase PAPase was chosen from among other candidate acid phosphatases, based on its upregulation under N deprivation in the transcriptomics study. Its expression increased progressively under all conditions, but most noticeably under N deprivation. The *PAPase* transcript levels dropped sharply after nutrient replenishment, suggesting that this isoform may be involved in polyP hydrolysis under N deprivation and in the control culture, in the stationary phase. *VTC4* putatively encodes a polyP polymerase subunit of the vacuolar transporter chaperone complex (VTC). VTC4 is involved in polyP synthesis in acidocalcisomes, and microautophagy in yeast (Livermore et al., 2016), but its function has not yet been studied genetically in photosynthetic organisms (Sanz-Luque et al., 2020). *VTC4* showed a different expression pattern under P and N deprivation, attaining maximal levels after 2 and 5 days, respectively. Notably, in *C. reinhardtii*, *VTC4* expression also attained its maximum by day 5 of N deprivation and then decreased, supposedly associated with polyP synthesis under N starvation (Goodenough et al., 2019). *VTC4* expression increased only after nutrient resupply to P-deprived cells, likely to circumvent the consequences of P deficiency through polyP re-synthesis. It is worth noting that the transient peak of expression of both *VTC4* and *PAP* was recorded after 12 h of N deprivation, which may indicate a compensatory effect under stress challenge.

The expression of the three genes involved in fatty acid catabolism by beta-oxidation (ABC-transporter D family member, ABC; multifunctional protein, MFP; and 3-ketoacyl-CoA thiolase, KAT) was monitored. The expression patterns of *KAT* and *MFP* – the homologs of plant and algal peroxisomal counterparts – were similar to those of *MLDP* and *ATG8*, although they were expressed at a lower level. Three genes showed a different expression pattern upon nutrient replenishment: the abundance of *MFP* and *KAT* transcripts increased only after nutrient resupply to the P-starved and control cells. Consistent with the cessation of the transcriptional activation of ARA and TAG biosynthesis after nutrient resupply, *DesD5*, *MLDP*, and *ATG8* showed a similar drop in expression after nutrient resupply. At the same time, the transcriptional regulation of the examined β-oxidation genes was not apparent, as indicated by the increase in *KAT* expression only.

### ATG8 Protein Levels Under Nutrient Deprivation and After Nutrient Resupply

**Figure 9:**
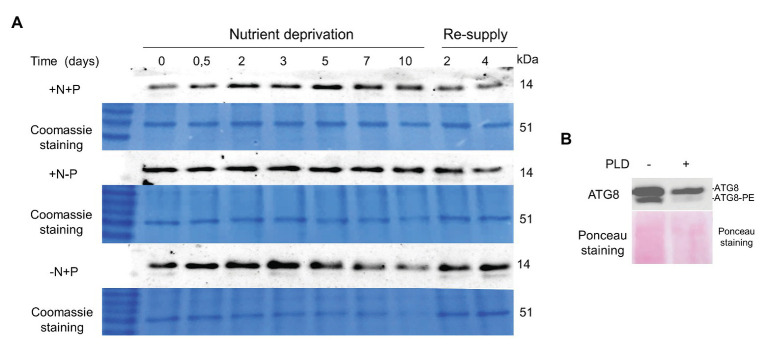
ATG8 levels in the replete control (+N+P), during P (+N−P), N (−N+P) deprivation and following nutrient resupply. **(A)** Time-course of ATG8 production in the control culture (+N+P), during P (+N−P) and N (−N+P) deprivation detected by western blot analysis. Re-supply: on day 10, cells from all treatments were harvested and resuspended in a complete nutrient-replete medium for growth recovery. Then, 2 μg of total protein was resolved on 15% SDS-PAGE gel. A section of the gel stained with Coomassie brilliant blue is shown as a loading control. **(B)** Phospholipase D treatment of protein sample 2 days after nutrient re-supply to N-starved cells (shown in **A**). Approximately 20 μg of protein was treated with 100 units of cabbage phospholipase D (PLD; Sigma) for 1 h at 30°C, and resolved on the 15% SDS-PAGE gel with 6 M urea. The loading control is the nitrocellulose membrane stained with Ponceau S, Red.

The *ATG8* gene showed a prominent upregulation during nutrient deprivation. The changes in ATG8 protein production during N and P deprivation were investigated using immunoblotting with the anti-CrAtg8 antibody (Figure 9). The ATG8 protein was detected in N-replete cells at the experiment onset (Time-0 for all conditions), and attained the maximal level after 5 days of replete conditions, consistent with the depletion of the N source (Kokabi et al., 2019). We notice a technical issue with ATG8 detection at Time-0 in the control (+N+P lane), since the same loading for Time-0 was done for N- and P-deprived samples. Under N deprivation, the ATG8 abundance increased, attaining a maximal level at day 3, followed by a gradual decline. In contrast to N deprivation, ATG8 remained literally unaltered under P deprivation. ATG8 production increased after nutrient resupply to N-starved cells, but not in other treatments. A weak cross-reactive band below the main ATG8 band was observed under N deprivation and after nutrient replenishment. To test whether this may represent a conjugated ATG-PE form, which is more hydrophobic than free ATG8, the phospholipase D (PLD) treatment was carried out on a concentrated protein sample of the nutrient-deprived (−N+P) cells 2 days after nutrient resupply (Figure 9B). The band with the faster electrophoretic mobility became less prominent after the PLD treatment, suggesting that it may represent the PE-conjugated form.

### Pharmaceutical Treatment With Concanamycin A Impairs ARA Biosynthesis

**Figure 10:**
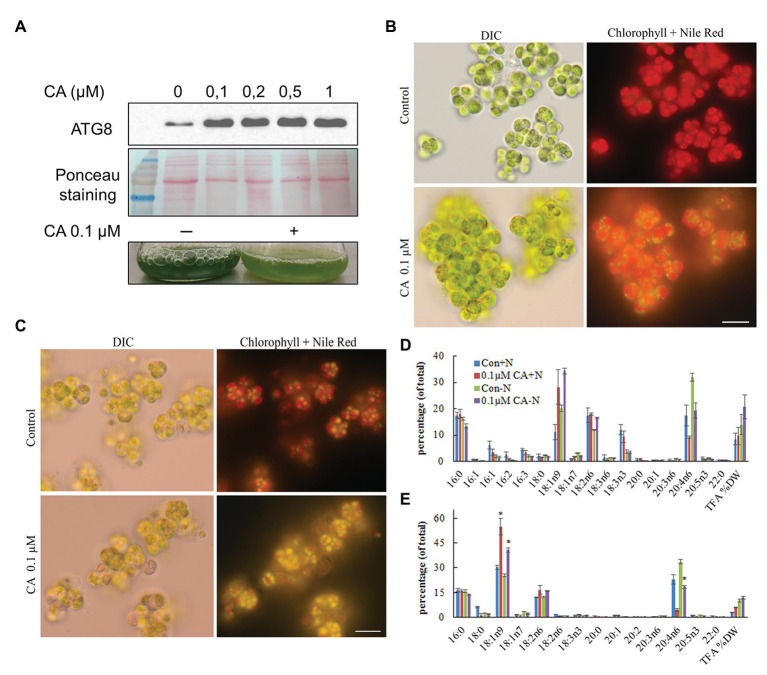
Effect of Concamycin A (CA) on the growth and accumulation of arachidonic acid in *L. incisa*. **(A)** CA treatment promotes ATG8 accumulation in N-replete cells. CA was applied to N-replete cells in a 12-well plate for 12 h at the indicated concentration range; 2 μg of total protein extract was resolved on a 15% SDS-Urea gel. Micrographs of *L. incisa* cells 3 days after treatment of nutrient-replete **(B)** and N-deplete **(C)** cultures with 0.1 µM Concamycin A, compared to respective controls. DIC: Differential interference contrast; Lipid droplets stained with Nile Red are seen in bright yellow; Chloroplast area is seen as a red fluorescent area; Scale bars 10 µm. **(D)** Quantification of total fatty acids in cell biomass, and **(E)** TAG in *L. incisa* treated with 0.1 μM CA under nutrient repletion and N deprivation, compared with the respective controls. Cells were sampled after 3 days of indicated conditions. The data represent a mean ± SD from two biological replicates, each analyzed with three analytical repeats. Statistical analysis was carried out using Student’s *t*-test (^*^ denotes a significant difference from untreated control, *p* < 0.05).

Autophagy inhibitors are a valuable tool to experimentally link lipid metabolism and autophagy. Concanamycin A (CA) inhibits the activity of vacuolar H^+^-ATPases, thereby inactivating vacuolar protein hydrolases and disrupting autophagic flux (Yoshimoto et al., 2004; Thompson et al., 2005). As a result of suppressing autophagic degradation, both ATG8 and its ATG8-PE lipidated form accumulate. CA was applied in N-replete cultures at a concentration range that had proved effective in *C. reinhardtii* (Couso et al., 2018). The ATG8 protein accumulated in a dose-dependent manner with the CA treatment (Figure 10A), suggesting suppression of autophagic flux. However, we did not detect a faster-moving band that could have been attributed to lipidated ATG8-PE, despite applying a 6 M Urea-15% SDS-gel, which allows separation of the free and PE-conjugated ATG8 forms. Hence, ATG8 lipidation in *L. incisa* remains enigmatic and needs further detailed investigation, beyond the scope of this work.

We next asked whether CA treatment would affect the dynamics of photosynthetic pigments, and TAG accumulation and composition. In N-replete flask-cultures, 0.1 μM of CA caused strong growth inhibition, which was observed as the cessation of Chl production and bleaching of the cultures (Figure 10A). It should be noted that cell counting to record growth curves is not feasible in *L. incisa*, because it forms clusters of multiple attached daughter cells. Microscopic observation revealed LD formation in replete cells under CA treatment (Figure 10B). In contrast, 0.1 μM of CA applied to N-deprived cultures did not exhibit a noticeable effect on culture performance and cell morphology (Figure 10C). Photosynthetic pigments degraded to a similar extent, accompanied by visually undisturbed LD formation. Importantly, total FA and TAG analysis showed that CA caused a strong increase in 18:1*n*9, the early precursor of ARA in the omega-6 pathway of LC-PUFA biosynthesis (Bigogno et al., 2002), with a concomitant decrease in ARA proportion (Figures 10D,E). While the proportion of ARA declined, the content of TAG in the biomass slightly increased, indicating that autophagic flux is important for ARA accumulation in TAG.

**Figure 11:**
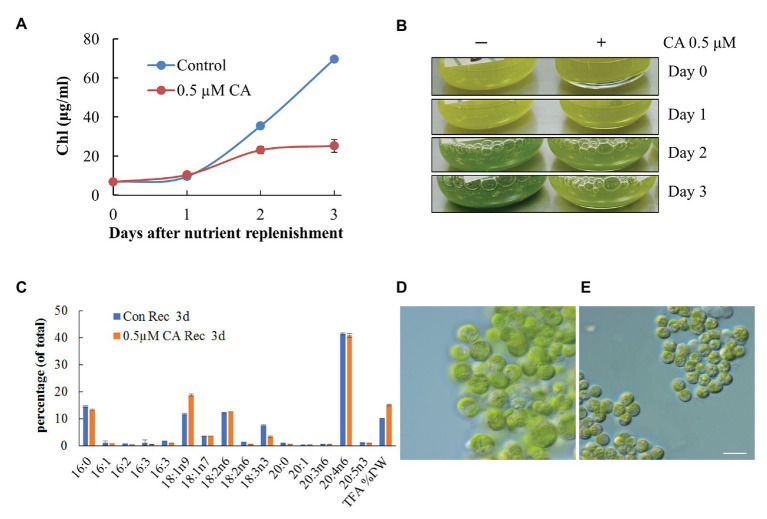
Concamycin A (0.5 μM) inhibits culture recovery after a shift from an N-deprived to an N-replete medium. Chlorophyll production **(A,B)**, total fatty acid composition and content **(C)**, and micrographs of control untreated **(D)** and treated cells **(E)** 3 days after nutrient replenishment to N-deprived cells with and without 0.5 μM CA. The data shown represent a mean ± SD from two biological replicates analyzed in three analytical repeats. Scale bars 10 µm.

To test whether CA affects TAG remobilization and growth recovery after nutrient resupply to N-deprived cells, we applied the inhibitor to cells transferred to an N-replete complete medium after 10 days of N deprivation. At 0.1 μM, CA did not affect culture recovery (not shown), but when applied at 0.5 μM, it strongly inhibited re-growth and the restoration of Chl production (Figure 11A). The impaired build-up of the photosynthetic apparatus under CA treatment was consistent with the results of microscopy and cellular fatty acid analysis, showing a higher total fatty acid content, a higher proportion of 18:1*n*9, and a decreased proportion of 18:3*n*3, 3 days after nutrient replenishment (Figure 11B). The latter fatty acid is associated with the major chloroplast glycerolipids and, thus, its decreased proportion indicates a lesser extent of photosynthesis system recovery. Consistent with the results of previous studies, the proportion of ARA in TAG did not swiftly decline following nutrient replenishment (Kokabi et al., 2019). Microscopic observation (Figures 11C,D) revealed that CA treatment repressed cell enlargement, division and proliferation, and recovery of the photosynthetic apparatus, and delayed LD degradation. These results suggest that autophagic flux may be involved in photosynthetic apparatus recovery and TAG mobilization.

### Expression of GFP-ATG8 Fusion in *L. incisa* to Monitor Autophagic Flux

**Figure 12:**
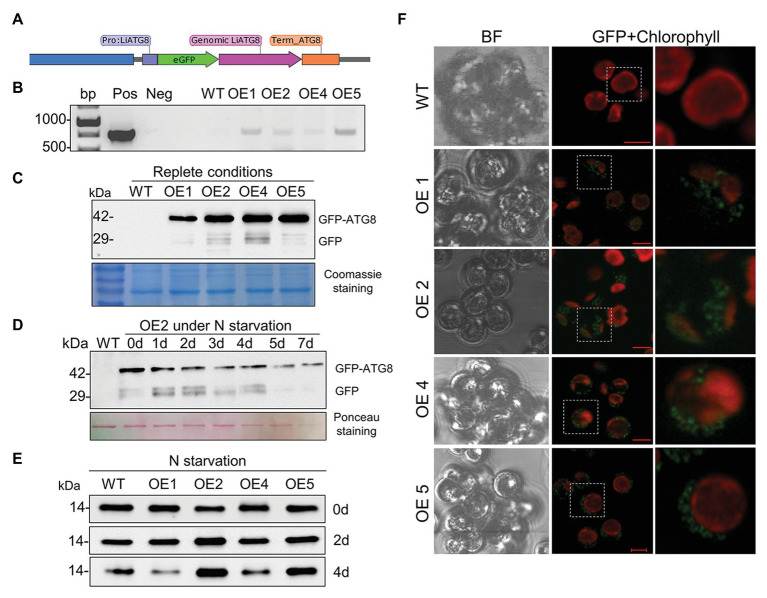
The establishment of the GFP-ATG8 autophagy marker in *L. incisa*. **(A)** Schematics of the plasmid construct pLiATG8::GFP-ATG8; **(B)** PCR validation of transgene integration using GFP-specific primers; **(C)** detection of GFP-ATG8 fusion protein and free GFP in OE lines under nutrient-replete conditions, using western blot analysis with the anti-GFP antibody. Coomassie blue-stained SDS-PAGE is shown below the blots as a loading control. **(D)** Detection of GFP-ATG8 and free GFP in OE2 line under N deprivation (−N+P) by immunoblotting with anti-GFP antibodies. Ponceau staining of the membrane of total proteins is shown as a loading control. **(E)** Immunoblotting with anti-ATG8 antibody for ATG8 detection in WT and transformant OE lines under N deprivation. **(F)** Visualization of the GFP signal in selected transformants using confocal microscopy. Scale bars = 5 μm.

Suppression of the vacuolar proteolytic function by CA caused accumulation of the marker autophagy protein ATG8 in replete cells (Figure 10A) and inhibition of ARA accumulation in TAG (Figure 10E), suggesting the involvement of autophagic flux. A plasmid construct carrying the GFP-ATG8 fusion was generated to assess the proteolytic cleavage of N-terminal GFP, a key assay for the detection of autophagic flux. The *LiATG8* genomic sequence was amplified with its promotor and terminator sequences (1,570 bp), fused at the N terminus with *eGFP*, and expressed in *L. incisa* by electroporation (Figure 12A). Transgenic lines were selected based on resistance to the herbicide SMM (Supplementary Figure S4A), conferred by the expression of the mutated form of the *AHAS* gene (Grundman et al., 2012; see Materials and Methods). Integration of *GFP* was verified by PCR in several transformants (Figure 12B); GFP-ATG8 protein expression was confirmed by western blotting with anti-GFP antibody (Figure 12C) and confocal microscopy (Figure 12F). Two out of the four selected transformants displayed stronger ATG8 accumulation after 2 and 4 days of N deprivation, detected by western blotting with anti-ATG8 antibody (Figure 12E). Using the GFP-antibody, the production of GFP-ATG8 fusion (the predicted molecular weight about 42 kDa), and the release of free GFP were detected in replete cells, suggesting its proteolytic cleavage. The release of GFP from the GFP-ATG8 fusion was also detected under N deprivation in the selected transformant (Figure 12D). The fluorescent signal of GFP was observed predominantly as circular structures dispersed in the cytoplasm of the transformed cells, which is consistent with the distribution of vacuoles in replete cells, suggesting that GFP may be delivered to the vacuoles. The transformant OE4 was subjected to different treatments to visualize the cellular localization of the GFP signal, 12 h after exposure to adverse conditions (P and N deprivation, and H_2_O_2_ treatment; Supplementary Figure S4B). However, the GFP signal stayed unvaried regardless the stress treatment applied. Furthermore, the substantial GFP signal observed in the untreated nutrient-replete cells of the transformants limited this approach for studying the enhancement of autophagy. Further in-detail characterization of the transformants is important, but it was beyond the scope of this study.

## Discussion

Different scenarios of transcriptome, proteome, and metabolome reorganization are associated with the differential rearrangement of lipid metabolism under N and P deprivation (López García de Lomana et al., 2015; Alipanah et al., 2018; Wördenweber et al., 2018; Kokabi et al., 2019). In this study, we aimed to provide a more detailed picture of *L. incisa* responses to N and P deprivation. It was hypothesized that the metabolic and energetic costs associated with enhanced ARA biosynthesis, in *L. incisa* under N deprivation, require increased catabolic activity and autophagy.

The observed changes in the TAG profile under N deprivation were consistent with the enhanced formation of ARA-containing species (Bigogno et al., 2002). In contrast, C18-containing TAGs were enhanced under P deprivation. The results of comparative lipid profiling of *L. incisa* indicated intensive membrane lipid breakdown under N deprivation compared with P deprivation. Under N deprivation, membrane lipid turnover and breakdown and neo-synthesis supply fatty acids for TAG assembly (Li et al., 2012; Rismani-Yazdi et al., 2012). Under P deprivation, membrane lipids are remodeled, whereby DAG, liberated from phospholipids, is used for non-phosphorous glycerolipid synthesis, while the deacylated G-3-P can be reused for TAG biosynthesis (Mühlroth et al., 2017). The increased ratio of two major galactolipids, DGDG to MGDG, is central for chloroplast membrane homeostasis under stress (Kobayashi, 2016). Accordingly, under N deprivation, the abundance of DGDG species decreased less than MGDG, while the abundance of lyso-DGDG species increased. The N-deprivation-induced release of acyl groups from the plastidial galactolipids, with the concomitant increase in the lyso-lipids, may be mediated by galactolipase/s (Li et al., 2012; Du et al., 2018) or the phospholipid: diacylglycerol acyltransferase (PDAT), which may act on different membrane lipids (Yoon et al., 2012; Liu et al., 2016). The liberation of chloroplast-derived fatty acids (linoleic and linolenic) and their volatile derivatives (fatty alcohols, fatty aldehydes, etc.), which could limit their integration into TAG, has recently been documented as an early response of *L. incisa* to N deprivation, consistent with the enhanced degradation of chloroplast glycerolipids and the transcriptional upregulation of putative galactolipid lipases (Kumari et al., 2020). Chloroplast dismantling is a characteristic consequence of N deprivation in microalgae whereby autophagy-mediated degradation of chloroplast components may play important roles in N scavenging, and the supply of amino acids and building blocks for enhanced FA and TAG biosynthesis.

Numerous acidic vacuoles with various inclusions are found in *L. incisa* cells (Watanabe et al., 1996; Merzlyak et al., 2007; Kokabi et al., 2019). We asked whether these organelles could be involved in the autophagic degradation and recycling of cellular components under nutrient deprivation. Double-membrane autophagosomes and autophagic vacuoles with a variety of cargo material were observed in microalgae (Ramundo et al., 2014; Schatz et al., 2014; Lütz-Meindl, 2016). Although we did not observe canonical autophagosomes in *L. incisa*, we observed some manifestations of autophagic activity in both nutrient-replete and deprived cells in this and a previous study (Kokabi et al., 2019). Autophagic vacuoles were observed under N deprivation, as well as the production of the key autophagy protein ATG8 in replete cells and under N and P deprivation.

The membrane-bounded acidic organelles, acidocalcisomes, play a key role in autophagy in eukaryotic microbial parasites (Li and He, 2014; Docampo, 2016). The role of acidocalcisomes in autophagy in microalgae remains to be investigated. In microalgae, acidocalcisomes are involved in the sequestration of nitrogenous compounds and polyP in close association with the uptake of inorganic cations (Ca^2+^, Fe^2+^, Mn^2+^, Mg^2+^, and others; Pick et al., 1990; Ruiz et al., 2001; Goodson et al., 2011; Moudříková et al., 2017; Shebanova et al., 2017; Tsednee et al., 2019). Although polyP and nitrogenous compounds were detected in *L. incisa*, in the acidic vacuoles with granular bodies (Kokabi et al., 2019), Ca^2+^ was not detected by the EDX analysis, applied in this and our prior study. In *C. reinhardtii*, acidocalsomes appeared to be restricted to stationary and N-starved cells, and can be distinguished from lytic vacuoles based on the membrane fracture-face morphology (Goodenough et al., 2019). In *L. incisa*, however, the organelles with distinct granular bodies were more abundant in nutrient-replete cells (Watanabe et al., 1996; Merzlyak et al., 2007; Kokabi et al., 2019). To ascertain their composition and structure in *L. incisa*, advanced microscopic methods have to be applied.

The genome of *L. incisa* contains a set of putative ATG-related genes consistent with bioinformatics studies in other microalgae (Jiang et al., 2012; Shemi et al., 2015; Zienkiewicz et al., 2020). Some of the *L. incisa* ATG proteins displayed additional structural features compared to their counterparts outside of Chlorophyta. WD40 repeats, which have been reported in some algal ATGs (Shemi et al., 2015), were found at the carboxyl terminus of ATG16 and VPS15 in several tandem copies. This suggests that WD40 domains with the ability to scaffold and interact with a variety of cellular components (Smith, 2008) may endow these proteins with additional functions, which warrant further investigation.

Autophagy-related genes are upregulated by different stresses in microalgae, specifically under TAG-inducing conditions (Perez-Perez et al., 2012; Goodenough et al., 2014; Ramundo and Rochaix, 2014; Zienkiewicz et al., 2020). In *L. incisa*, among several ATG genes that were upregulated under N deprivation, *ATG8* was upregulated particularly strongly. The key autophagy protein was produced and the gene expressed in replete cells, consistent with the notion of basal autophagy activity. The expression patterns of *ATG8*, *MLDP*, and *DesD5* under N deprivation closely mirrored each other, pointing to the relationship between autophagy, LD biogenesis, and ARA sequestration in LDs in N-deprived *L. incisa*. Under P deprivation, the *DesD5* transcript and ATG8 protein displayed stable levels, but an increasing pattern was determined in the *ATG8* transcript level. This may suggest a different mechanism involved in P-deprivation-mediated signaling in autophagy-related genes. It has recently been shown that P limitation signaling and autophagy in *C. reinhardtii* are regulated by the TORC1’s component LST8 and the upstream transcription factor PSR1 (Couso et al., 2019). The enhancement of autophagy under N deprivation allows for the degradation, recycling, and reutilization of cellular components and reserves, consistent with the results on the dynamics of ATG8 protein and gene expression and TEM of N-deprived *L. incisa* cells.

When LD mobilization was induced by nutrient resupply to N-deprived cells, the ATG8 protein’s production was inconsistent with the *ATG8* gene expression. The observed discrepancy can be explained by the sufficient amount of transcript at the early stages of recovery. It is also possible that the turnover rate of the transcripts exceeded the rate of synthesis and/or post-transcriptional modifications were involved. It is also plausible that transcripts are present but sequestrated or frozen by proteins that keep them stable and hinder translation. Alternatively, LD degradation may be mediated by ATG8-independent mechanisms, such as microautophagy and lipolysis, in agreement with the identification of TAG lipases in the LD proteome of *L. incisa* (Siegler et al., 2017) and the previous study on remobilization of TAGs in *L. incisa* (Khozin-Goldberg et al., 2005).

Despite significant progress, relatively little is yet known about the molecular mechanisms underlying autophagy and its interaction with LD formation and remobilization in microalgae. The induction of autophagy by inhibition of the major nutrient sensor kinase TOR triggers autophagy in microalgae and LD accumulation without applying stress stimuli (Imamura et al., 2015; Mukaida et al., 2016; Prioretti et al., 2017). Class III PI3K plays a major role in regulating lipid metabolism and autophagosome formation, and its inhibition or knockdown enhances lipid content and TAG formation (Ramanan et al., 2018; Zienkiewicz et al., 2020). However, there are claims that TOR activation can regulate TAG biogenesis without activating the autophagic pathway (Kajikawa and Fukuzawa, 2020). Further, LDs are frequently found in proximity to, or even inside, the vacuoles (Gorelova et al., 2014; Tsai et al., 2017; Zienkiewicz et al., 2020), which is consistent with the TEM observations of this work. However, LD formation did not appear impaired in the *atg* mutants of *Chlamydomonas*, which displayed compromised recycling of various cytoplasmic components (Kajikawa et al., 2019), suggesting alternative ways of interacting.

The pharmaceutical inhibition of the lytic vacuolar function arrested growth and induced LD formation in replete cells of *L. incisa*; the effect is similar to the inhibition of the earlier steps of autophagy in other studies. In *Chlamydomonas*, CA suppressed formation of LDs and TAG accumulation under N and P deprivation (Couso et al., 2018). In *L. incisa*, CA did not enhance deleterious effects on the photosynthetic pigment degradation and LD formation under N deprivation. However, it led to a significant decrease in the ARA level in TAG. These findings suggest that autophagic flux may be particularly important for sustaining ARA biosynthesis under N deprivation. Since, ARA biosynthesis from 18:1 involves three energy-requiring desaturation and one elongation steps, the plausible explanation is that autophagy-mediated degradation of cellular components supports expenditures associated with the enhanced ARA biosynthesis.

LD mobilization after nutrient resupply provides fatty acids for restoring and rebuilding photosynthetic machinery and cellular membranes (Khozin-Goldberg et al., 2005), as well as a source of energy to fuel growth recovery. We observed changes in the osmophilicity of the LD-core during TAG remobilization, possibly attributed to the consumption of certain TAG species. LDs may indeed exhibit different internal organizations, as well as organelle associations, depending on cellular states (Mahamid et al., 2019). The EDX analysis pointing to LDs showed a high Ca^2+^ signal associated with the degrading LDs. Caleosin is a Ca-binding oil body/LD surface protein, whose role in seed oil-body degradation and interaction with vacuoles during seed germination is well-established (Poxleitner et al., 2006). We can speculate that LD-associated caleosins may play a role in LD degradation during growth recovery, mediating the degradation of fatty acids released from LDs. It is also plausible that caleosin may function in the maintenance of LDs under deprivation (Siegler et al., 2017).

To conclude, we provide several pieces of evidence that highlight the homeostatic role of autophagy in proliferating *L. incisa* cells and the role played by autophagy under N deprivation. We suggest that the ARA-rich TAG biosynthesis, as an energy- and resource-demanding challenge for N-deprived cells, has to be supported by numerous metabolic changes to meet these demands, including increased catabolic activity and autophagy. Although this microalga is difficult to transform, we managed to obtain the GFP-ATG8 expressing lines. Pharmaceutical inhibition of the late stages of autophagy halted nutrient-replete growth and reduced ARA accumulation in TAG under N deprivation, as well as impaired the buildup of photosynthetic membranes and delayed LD degradation after nutrient replenishment. Further investigation is obviously needed to elucidate the molecular basis of autophagy and LD degradation machinery, and LD-autophagy interaction in *L. incisa*.

## Data Availability Statement

The raw data supporting the conclusions of this article will be made available by the authors, without undue reservation.

## Author Contributions

KK and IK-G conceived the study and analyzed the data and wrote the manuscript. KK performed the experimental work. BZ designed the expression vectors and conceived the transformation of *L. incisa*. OG and AS performed the TEM and elemental analysis with TEM-EDX at the User Facilities Center of M.V. Lomonosov Moscow State University. SD-C performed TAG and fatty acid analysis. MI and SM performed the lipidomics analysis. IK-G and SB were involved in funding acquisition. All authors read, reviewed, and approved the manuscript.

### Conflict of Interest

The authors declare that the research was conducted in the absence of any commercial or financial relationships that could be construed as a potential conflict of interest.

## Footnotes

^1^repeat.biol.ucy.ac.cy/iLIR

^2^bioinformatics.psb.ugent.be/hfAIM

^3^elm.eu.org
