# Supplementary material for: Lipidome Remodeling and Autophagic Respose in the Arachidonic-Acid-Rich Microalga Lobosphaera incisa Under Nitrogen and Phosphorous Deprivation
Source: Front Plant Sci. 2020 Nov 27;11:614846. doi: 10.3389/fpls.2020.614846 (PMC7728692; doi:10.3389/fpls.2020.614846)
Supplement: Supplementary file 1 [file Data_Sheet_1.pdf]

Supplementary material

Lipidome remodeling and autophagic response in the arachidonic-acid-rich microalga *Lobosphaera incisa* under nitrogen and phosphorous deprivation

Kamilya Kokabi[1,2], Olga Gorelova[3], Boris Zorin[2], Shoshana Didi-Cohen[2], Maxim Itkin[4], Sergey Malitsky[4], Alexei Solovchenko[3,5,6], Sammy Boussiba[2], Inna Khozin-Goldberg[2]*

[1]The Albert Katz International School for Desert Studies; The Jacob Blaustein Institutes for Desert Research, Ben-Gurion University of the Negev, Midreshet Ben-Gurion 8499000

[2]Microalgal Biotechnology Laboratory, The French Associates Institute for Agriculture and Biotechnology of Drylands, The J. Blaustein Institutes for Desert Research, Ben-Gurion University of the Negev, Sede Boqer Campus, Midreshet Ben-Gurion 8499000, Israel

[3]Department of Bioengineering, Faculty of Biology, Moscow State University, GSP-1, Moscow 119234, Russia

[4]Metabolic Profiling Unit, Life Science Core Facilities, Weizmann Institute of Science, Rehovot, Israel

[5]Institute of Natural Sciences, Derzhavin Tambov State University, 392000 Tambov, Russia

[6]Peoples Friendship University of Russia (RUDN University), Moscow, Russia

[#]Present address: Weizmann Institute of Science, Rehovot, Israel

[‡]Correspondence: e-mail: khozin@bgu.ac.il; Tel.: +97286563478

Supplementary Table 1. Primers used in this study

Gene symbol and number	Forward primer sequence 5' to 3'	Reverse primer sequence 5' to 3'
**Primers used in qRT-PCR**		
SNRP_ g7308	ACAGTGGACGAGAAGATGAAGACAGA	GCAGATGCTGCCTGTCTAACAC
COX3_g10280	GCAGGTTAACCATGCAGAGGAGG	GCGTGTGACAGGTCATACACCG
PAPse_g13792	AGTACGATCAGAAGCAGGTGGC	TTGTAGTCCAGCTGATGCAGGG
VTC4_g13494	TACCTGGGCTTCCACAAGATCC	CTCTCATACTGGAAGACGGGCA
ABC_g1747	CAAGACGCTGCTGGCATACA	CGGTCCACGTAGTTCAGCTTGTAGT
KAT_g8556	GCTGGAGACAACTCTGGCTATG	CGGCAGGTGTGTCCTTCAAA
MFP_g12483	GTGAGGGCTATCGTCATAACAGG	CGAGCACAGTTTGTCGTTGATG
MLDP_g555	ACTTCCGCCTACCAGTTCTA	CTTGTAGTGCTCCAGGTTCTTT
ATG8_g4496	GACAGAAGTCTTTCAAGGAGGAG	GATGTCTGGTATGTCGCTCTTT
DesD5_g4602	TAAGTGCCAGGGCTGTGCTAGA	GAACTGACCCTCCTCTGTGTCCT
VTC4_g13494	TACCTGGGCTTCCACAAGATCC	CTCTCATACTGGAAGACGGGCA
PAPse_g13792	AGTACGATCAGAAGCAGGTGGC	TTGTAGTCCAGCTGATGCAGGG
**Primers used in GFP-ATG8 expression plasmid construction**		
AHAS_3’	AAAGCGGCCGCAGGGAGCAGGTCATCAGGAAG	GCATGTGGAGCACGTGCTG
ATG8_SpeI	AAAACTAGTCGCCCCGTTTCGGGACAA	AAAACTAGTGGTCTGCGAACCCCATGTGT
ATG8_infusion	ATGGCTGCCAGACAGAAGTCT	GGCAGGACCTTCAGGCACTAG
GFP_infusion	CTAGTGCCTGAAGGTCCTGCCATGGCGAAGGGCGAGGAGCT	AGACTTCTGTCTGGCAGCCATCTTGTACAGCTCGTCCATGCCG
**Primers used for verification of plasmid integration**		
GFP-ATG8_seq	AAAGCCAGGCAGCAGTGTCTA	GCAGCCATCTTGTACAGCTCGT
**Primers used for gene amplification and verification**		
ATG3_g4126	ATGAGGAACGCTCTGCACAAC	TCACTCTATCGTTGGCACCACG
ATG7_g5114	ATGGCTAATAAGATGGTTCTGCAGT	TCACAAGCTCGTCCACTCGT

Supplementary Table 2. Putative ATG-related genes annotated in the genome of *Lobosphaera incisa*

	Gene abbreviation	Putative functional annotation	*Li*ATG genes	Description
ATG1 complex	ATG1	Autophagy-related protein 1	**g10441**	**804 aa**<br>STKc_ATG1_ULK_like N – terminal domain–containing protein with a long C-terminal extension, which occurs in Charophytes, Bryphytes and some green microalgae (Chlorophyta)
	ATG13	Autophagy-related protein 13	**g9857**	**1017 aa**<br>ATG13-N-terminal domain-containing protein (264 AA, proline-rich, disordered), with an extended C terminus. Most similar to ATG13 of Chlorellas.
	ATG101	Autophagy-related protein 101	**g4696**	**210 aa**<br>Homologous proteins in Chlorophytes, Charophytes, Bryophytes and higher plants
ATG9 complex	ATG9	Autophagy-related protein 9	**g6797**	**770 aa**<br>Membrane multi-path protein with 7 TMDs
	ATG2	Autophagy-related protein 2	**g11309**	**2145 aa**<br>Autophagy-related protein 2-domain in the C terminal part; 42% identity to *Chlorella variabilis,* 32% to *Arabidopsis thalians*, 24% i to the N-terminal region of yeast Chore or VPS13
	ATG18	Autophagy-related protein 18	**g11431**	**401 aa**<br>With a single WD40 repeat at 173-221 aa
	ATG18-like	Autophagy-related protein 18	**g11704**	**358 aa**<br>with 3 copies of WD40 repeat
	VMP1	Vacuole membrane protein 1	**g8948**	**438 aa**<br>Homologs in many sequenced Chlorophyta
PI3K III complex	ATG6	Autophagy-related protein 6/VPS30	**g1861**	**452 aa**
	ATG14	Autophagy-related protein 14	**g5843**	**504 aa**<br>ATG-14 domain-N-terminal -containing protein (~300 AA) with homologs in Chlorophyta and higher plants
	UV_resistance/ autophagy_Atg14	UV radiation resistance protein/autophagy-related protein 14	**g6478**	**782 aa**<br>Homologs in Chlorophyta and higher plants

	VPS15	Vacuolar Protein Sorting 15	**g14916**	**1476 aa**<br>N-terminal kinase domain, central heat domain, and several WD40 repeats at the C terminus as in other organisms, including *S. cerevisiae*
	VPS34	Vacuolar Protein Sorting 34	**g8565**	**867 aa**
	AMRA1	Activating molecule in BECN1-regulated autophagy protein 1	**g9351**	**1234 aa**<br>with 3 copies of WD-40 repeats, in the N terminus and in the C terminus
Two ubiquitin-like conjugation	ATG3	Autophagy-related protein 3	**g4126**	**310 aa**<br>Conserved protein
	ATG4	Autophagy-related protein 4	**g4008**	**413 aa**<br>Homologs are present in many sequenced Chlorophytes, the highest identity to Coccomyxa
	ATG5	Autophagy-related protein 5	**g10190**	**307 aa**<br>>35% identity to ATG5 of Chlorophytes and Arabidopsis; 25% identity to human and mouse ATG5
	ATG7	Autophagy-related protein 7	**g5114**	**698 aa**<br>>55% to *Chlorella variabilis* and *Coccomyx*a, ~ 50% to *Homo sapiens* and *Arabidopsis*
	ATG8	Autophagy-related protein 8	**g4496**	**120 aa**
	ATG10	Autophagy-related protein 10	**g12925**	**311 aa**
	ATG12	Autophagy-related protein 12	**g3729**	**91 aa**
	ATG16	Autophagy-related protein 16	**g6985**	**581 aa**<br>Autophagy-related-16 N-terminal domain, with multiple WD-40 domains in the C terminus (281-580 aa).This architecture is conserved in the green lineage.

Supplementary Table 3. Selected genes monitored in qRT-PCR and their expression levels determined in the transcriptomics study*. † FPKM - fragments per kilobase of transcript per million. ‡The significance of fold change is indicated by the *p* value adjusted for multiple testing at a false discovery rate of 0.05.

Gene ID	Gene symbol	FPKM†		Fold-change to time 0	Adjusted *p*-value‡
		d 0	d 3-N		
g555	MLDP	261.04	1211.61	4.98	0.044
g1747	ABC	24.96	20.85	0.77	0.888
g4496	ATG 8	264.77	962.46	4.14	0.027
g4602	DesD5	178.64	1177.8	7.29	0.013
g7308	SNRP	210.83	219.48	1.16	0.955
g8556	KAT	63.29	89.9	1.43	0.572
g10280	COX	295.07	293.39	1.07	0.979
g12483	MFP	74.98	138.28	1.99	0.322
g13494	VTC4	60.33	43.54	0.73	0.741
g13792	PAPase	41.98	122.99	3.28	0.017

*The genomic and transcriptomics data of *L. incisa* were generated as described in Siegler et al. (2017) and deposited in the NCBI GEO database under accession number GSE94666.

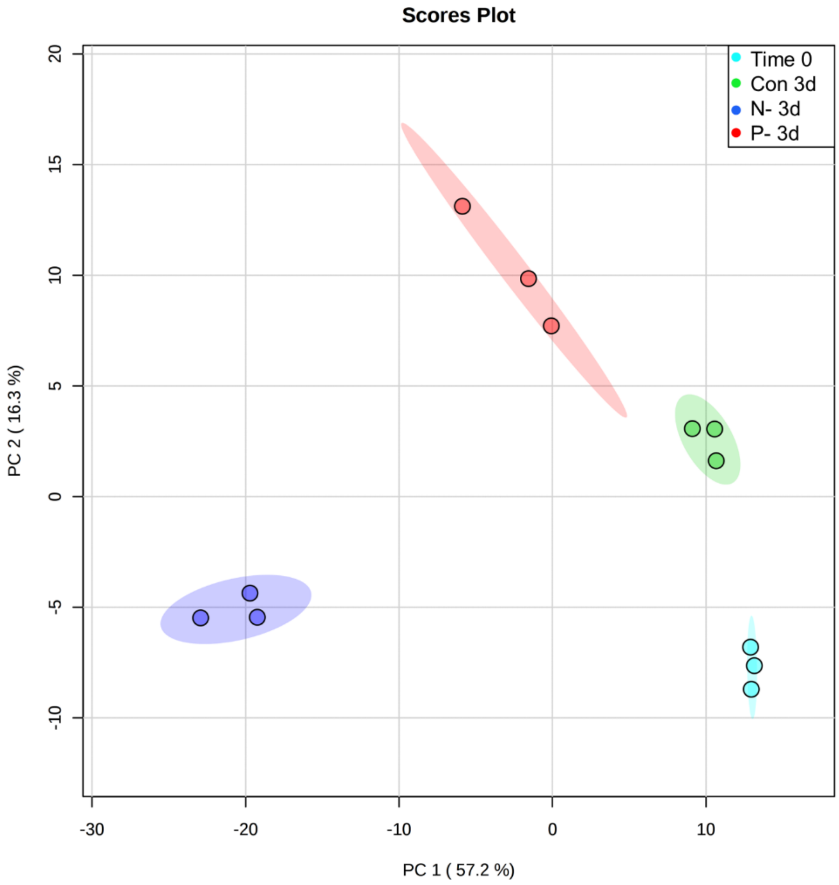

Supplementary Figure 1. Principal component analysis (PCA) of all lipid species detected by lipidomics analysis at the onset of the experiment (Time 0) and after 3 days of nutrient-replete (Con 3d) or depleted conditions (-N and -P). PCA showed a clear separation between treatments, explaining 73.5% variance (PC1+PC2). PCA scores plot was created in MetaboAnalyst 3.0 (Chong et al. 2018).

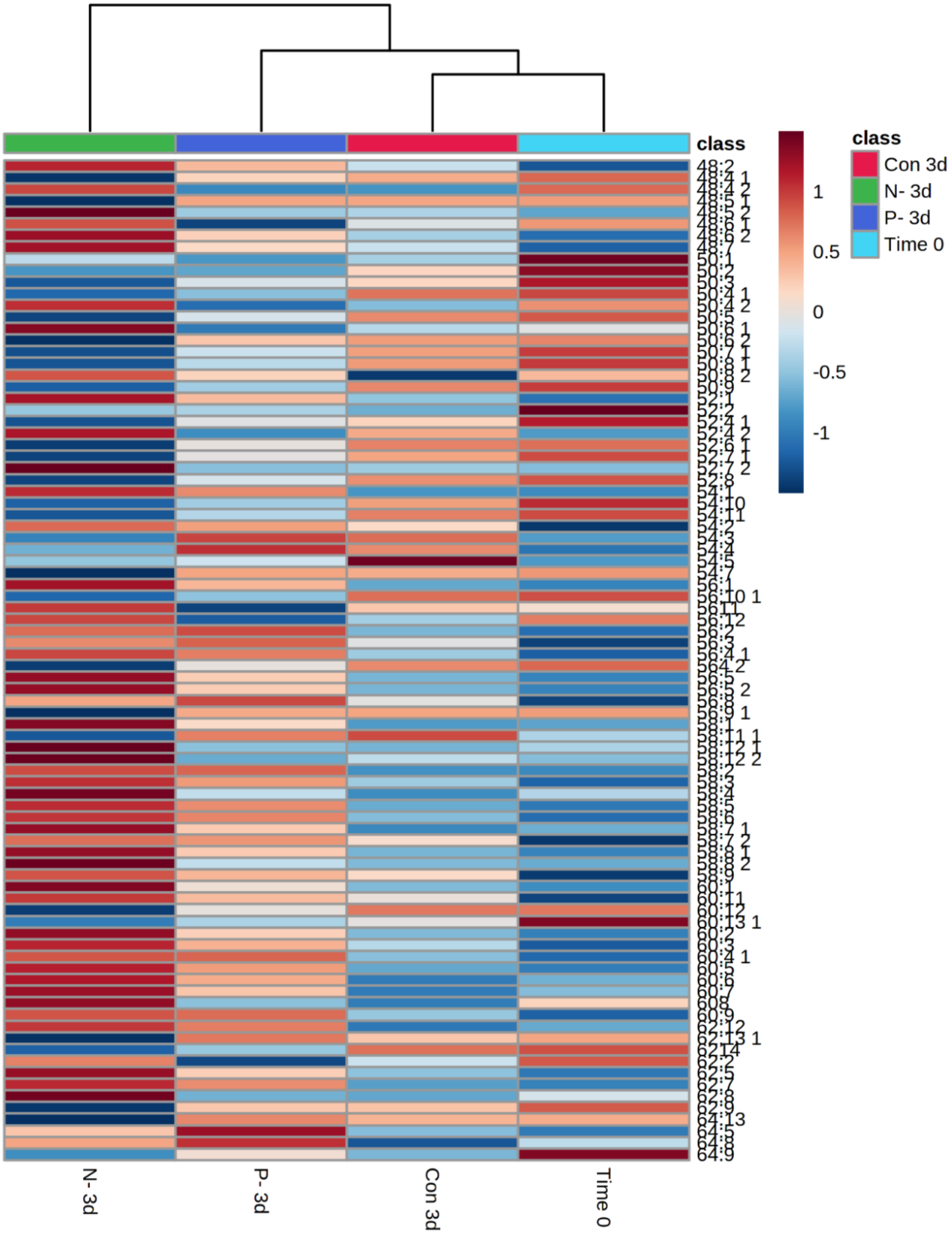

Supplementary Figure 2. Patterns in TAG species changes in *L. incisa* after 3 days of N and P deprivation. The results are shown for the top significantly different TAG species ranked by ANOVA according to the adjusted p-value (FDR) cutoff 0.05. Statistical **analysis and heatmaps** were performed at https://www.metaboanalyst.ca. The original data were quantile-normalized and log-transformed. Lipid species are designated as C:N, where C is the total number of carbons, and N is the total number of double bonds.

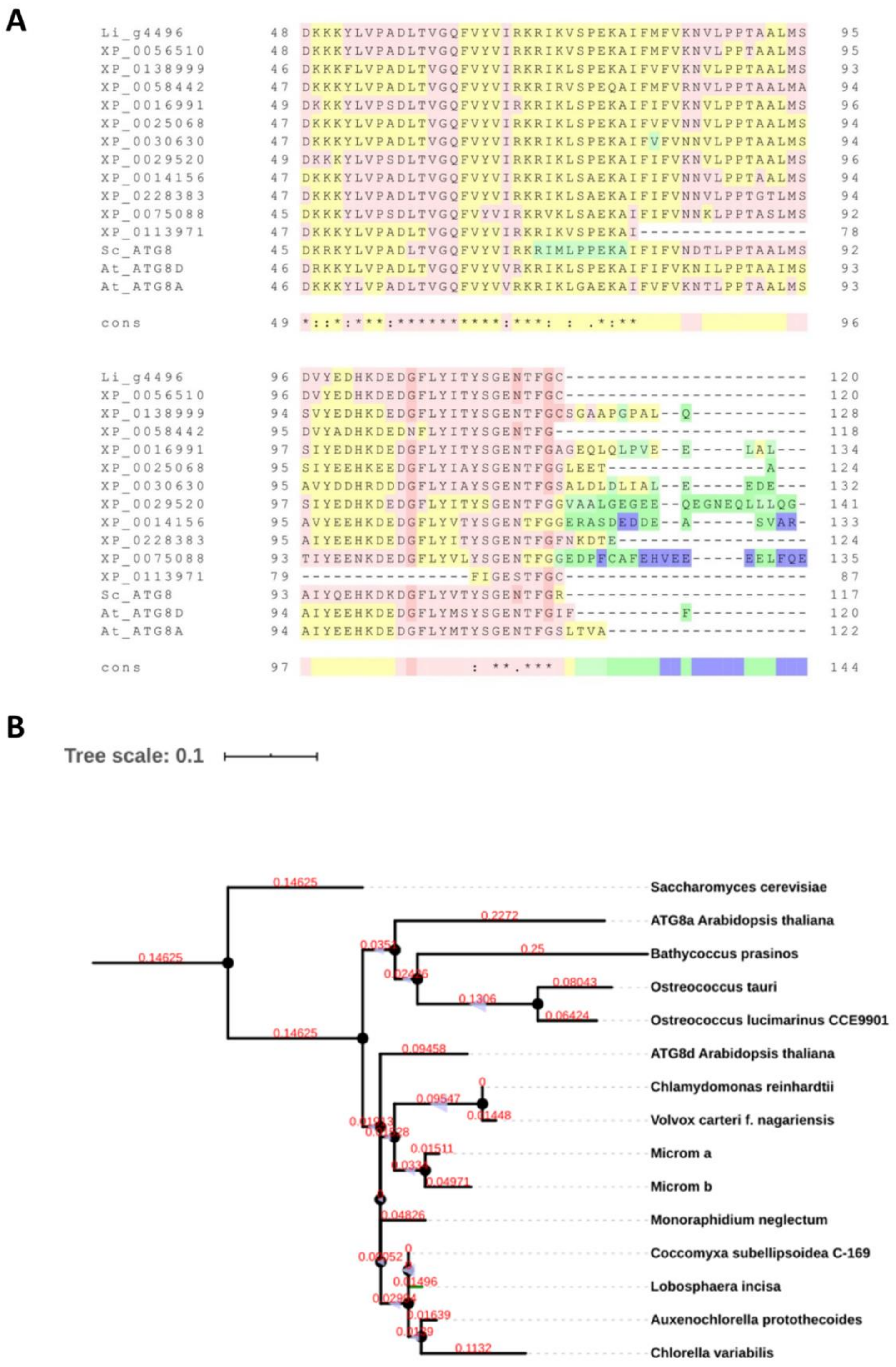

Supplementary Figure 3. Comparative analysis of the ATG8 protein from *Lobosphera incisa* and other organisms. A. Multiprotein sequence alignment of *Li*ATG8 against other members of the ATG8 family generated using T-Coffee (11.00.8cbe486) multiple alignment program at http://www.phylogeny.fr. The regions of high conservation are highlighted in red and yellow BAD AVG GOOD. *Li*_g4496 (*Lobosphaera incisa*), XP_0056510 (*Coccomyxa subellipsoide*a C-169), XP_0138999 (*Monoraphidium neglectum*), XP_0058442 (*Chlorella variabilis*), XP_0016991

(*Chlamydomonas reinhardtii*), XP_0025068 (*Micromonas commoda*), XP_0030630 (*Micromonas pusilla* CCMP1545), XP_0029520 (*Volvox carteri* f. *nagariensis)*, XP_0014156 (*Ostreococcus lucimarinus* CCE9901), XP_0228383 (*Ostreococcus tauri*), XP_0075088 (*Bathycoccus prasinos*), XP_0113971 (*Auxenochlorella protothecoides*), Sc_ATG8 (*Saccharomyces cerevisiae*), At_ATG8D (NP_178631_ATG8D_ *Arabidopsis thaliana*) and At_ATG8A (sp|Q8LEM4|ATG8A *Arabidopsis thaliana*). B. Phylogenetic tree for *Li*AT8. A tree was made with 100 bootstraps was constructed at the iTOL (https://itol.embl.de/); branch support values are shown in red.

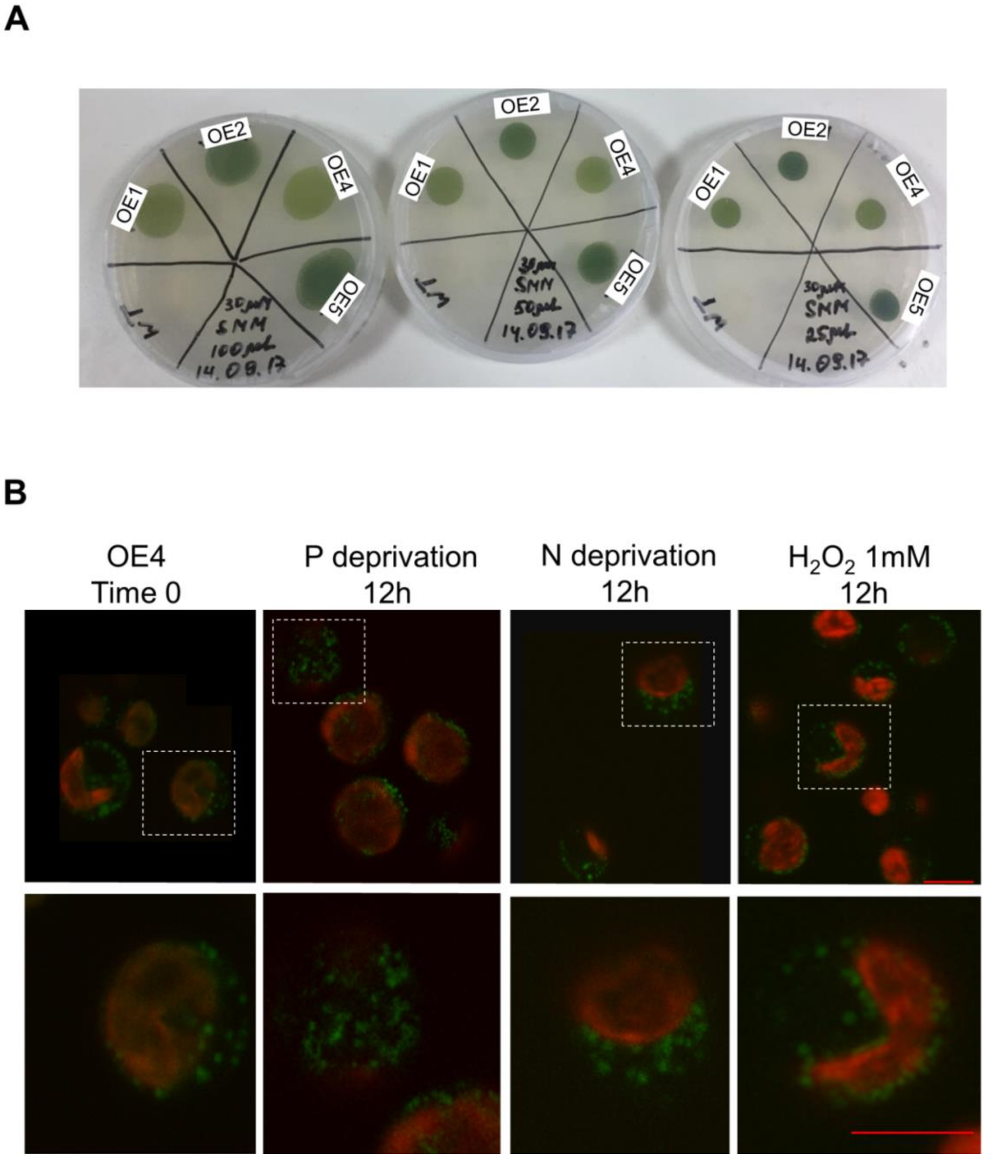

Supplementary Figure 4. Selection of GFP-ATG8 expressing transgenic lines (OE) based on SMM resistance, and confocal imaging of OE4 under different treatments. (A) Transgenic lines were selected based on resistance to the herbicide SMM applied in different concentrations. (B) Confocal micrographs of OE4 (GFP-ATG8 expressing transgenic line) 12 hours after different treatments. The demarked area in the upper panel is shown under higher magnification in the bottom panel. Scale bars 5 µm.
